# Pharmacogenomics-guided cardiovascular therapy in low- and middle-income countries: A systematic review of barriers, equity gaps, and pathways to personalized medicine

**DOI:** 10.1371/journal.pgph.0006591

**Published:** 2026-11-04

**Authors:** Md. Farid Reza Mashfi, Sumiya Sharmin Mou, Monalisa Riya, Nasiba Binte Bahar, Tasnuha Islam, Fayek Rafi Ahmad, Fabliha Fatima, Md. Elias Al-Mamun

**Affiliations:** 1 Department of Pharmacy, University of Asia Pacific, Dhaka, Bangladesh; 2 Department of Pharmaceutical Technology, Faculty of Pharmacy, University of Dhaka, Dhaka, Bangladesh; PLOS: Public Library of Science, UNITED STATES OF AMERICA

## Abstract

Cardiovascular disease causes more deaths than any other condition worldwide, and four in five of these deaths occur in low- and middle-income countries (LMICs). Pharmacogenomics (PGx) uses genetic testing to guide drug selection and administration, which can reduce adverse drug reactions and enhance treatment response; however, LMIC groups are significantly underrepresented in the evidence base, limiting equitable clinical translation. A systematic review was conducted, following PRISMA 2020 guidance, mapping PGx-guided cardiovascular therapy, implementation barriers, and equity gaps in LMICs. PubMed and Scopus were searched for studies published between January 2020 and December 2025 and included 24 studies (one randomized controlled trial, 19 observational studies, and four economic models); because of substantial clinical and methodological heterogeneity, findings were combined through SWIM guidelines, and certainty of evidence was assessed using GRADE. Genotype-guided warfarin dosing nearly quintupled the time patients spent in the therapeutic INR range compared with standard care, and decision-analytic models supported the cost-effectiveness of genotype-guided antiplatelet therapy. Ancestry-specific genetic variants relevant to heart-failure drug response, statin-induced myotoxicity, and resistant hypertension were identified, yet African-ancestry individuals made up less than 1% of global PGx study participants. The certainty of evidence varied from very low to moderate across outcomes, indicating a near-total dependence on observational designs and small sample sizes. High testing prices, workforce shortages, low physician genetic knowledge, and the lack of locally appropriate prescribing recommendations all remain obstacles to implementation. Based on these findings, a four-tiered, health-system-focused implementation approach is proposed that spans primary care to national policy. To create regionally calibrated prescribing guidelines in LMICs, multi-site randomized trials, ancestry-informed genomic data, and coordinated policy investment are required.

## Introduction

Cardiovascular diseases (CVDs) are the leading cause of death worldwide, leading to 20.5 million deaths in 2021, which is almost one-third of all deaths globally, with four in five of these deaths occurring in low- and middle-income countries (LMICs) [1]. Among the modifiable risk factors driving this burden, the prospective PURE study identified metabolic factors, principally hypertension and dyslipidemia, as leading contributors to CVD risk [2]. These risk factors are managed primarily through long-term pharmacotherapy, antihypertensive, and lipid-lowering agents, while anticoagulant and antiplatelet therapies govern outcomes once a cardiovascular event has occurred or recurrence must be prevented. Response to all these drug classes varies substantially between individuals, and much of that variability is genetic. Pharmacogenomics (PGx) addresses these issues directly by matching drug and dose to a patient’s genotype, yet the Sustainable Development Goal target of reducing premature deaths from non-communicable diseases remains off track in most countries, underscoring the need to ensure such prescribing reaches the populations that need it most [3].

Pharmacogenomics (PGx) is a promising technique for personalizing cardiovascular treatments to specific genetic profiles. Warfarin, a commonly used anticoagulant, has a narrow therapeutic index and significant inter-patient variability in dose requirements; common variants in CYP2C9, VKORC1, CYP4F2, and CYP2C genes account for roughly half of the dose variability [4], and suboptimal dosing can result in serious bleeding or thrombotic complications [4]. The majority of warfarin PGx studies and dose algorithms have been undertaken in White or Asian populations, raising concerns about the findings’ applicability to underrepresented groups and their potential to worsen health disparities [5]. For antiplatelet therapy, CYP2C19 catalyzes the bioactivation of the prodrug clopidogrel; individuals who are intermediate or poor metabolizers due to loss-of-function alleles have reduced platelet inhibition and an increased risk of major cardiovascular events, so genotype-guided selection of P2Y12 inhibitors is recommended [6].

However, the benefits of pharmacogenomics are unlikely to be distributed equitably. Pharmacogenetics is still developing and being clinically implemented in low- and middle-income countries [7]. Major barriers are lack of local genomic and outcomes data, and population-specific allele frequency information [8]; cost and inefficiency of genotyping methods, lack of standardized testing platforms and insufficient informatics infrastructure [7]; and policy, reimbursement, and human resource challenges, including privacy issues, limited regulatory frameworks, lack of biobanks and guidelines, poor clinician training, and lack of awareness and trust among healthcare professionals. The World Health Organization and other global health authorities emphasize that closing these gaps through regional biorepositories, local cost-effectiveness studies, and clear clinical guidelines for equitable implementation will be critical to accelerating genomics access in developing countries [8]. Evidence-based guidelines, such as the Clinical Pharmacogenetics Implementation Consortium (CPIC) recommendations for pharmacogenetics-guided warfarin prescription and CYP2C19-guided antiplatelet medicine, provide detailed decision-making assistance once genotype results are available [4,6].To address this gap, we conducted a systematic evaluation of LMIC-relevant material on PGx-guided cardiovascular therapy, identified implementation obstacles and equity gaps, and proposed alternatives to equitable precision cardiovascular care.

## Methods

### Protocol and registration

This review was conducted following a prespecified protocol and in accordance with the PRISMA 2020 guidelines [9]. The completed PRISMA 2020 checklist is provided in S1 PRISMA Checklist. The protocol was prospectively registered with the International Prospective Register of Systematic Reviews (PROSPERO; registration ID CRD420251150702) before study selection began to ensure transparency and reproducibility. The full review protocol is provided in S1 Protocol.

### Eligibility criteria

Predefined inclusion and exclusion criteria, structured using the PICOS (Population, Intervention, Comparator, Outcomes, and Study design) framework, were applied to determine study eligibility and are summarized in Table 1.

**Table 1:** PICOS eligibility criteria.

Element	Eligibility criteria
Population (P)	• Adolescents or adults receiving pharmacotherapy for cardiovascular diseases in LMICs (World Bank classification at time of study [10]) • Conditions: CAD, stroke/TIA, HF, AF, and related disorders • Multi-country or non-LMIC studies were eligible only when they addressed gene–drug pairs, ancestry groups, or implementation models judged directly transferable to LMIC populations or health-system contexts.
Intervention (I)	• PGx testing or genotype-guided therapy for cardiovascular drugs in LMICs. Examples: CYP2C19 (antiplatelets), CYP2C9/VKORC1 (warfarin), SLCO1B1 (statins), CYP2D6 (β-blockers)
Comparator (C)	• Standard/usual care without PGx guidance • Alternative strategies, or genotype-group comparisons (carriers vs non- carriers) • Implementation studies: before vs after PGx, or historical controls acceptable
Outcomes (O)	• Clinical efficacy: MACE, MI, stroke, stent thrombosis, hospitalization, all- cause mortality • Safety: major bleeding, myopathy, hepatotoxicity • Intermediate/biomarkers: platelet reactivity, INR/TTR, lipid changes, genotype-related dosing/process measures • Implementation: uptake, feasibility, turnaround time, cost-effectiveness, fidelity, equity of access • Equity/subgroups: differences by sex, SES, race/ethnicity, geography
Study design (S)	• Included: Randomized Controlled Trial / Controlled Clinical Trial / Pragmatic • Clinical Trial, Clinical Trial, Phase II–IV, Adaptive Clinical Trial, Equivalence Trial, Observational Study, Comparative Study, Multicenter Study (if comparative), Evaluation Study, Validation Study • Excluded: reviews/editorials/commentaries/abstracts; purely mechanistic genetics without clinical/implementation outcomes
Time frame	• Publications: 1 Jan 2020 – 31 Dec 2025
Language	English

In alignment with the registered review scope, LMIC relevance was defined as a priori to include research conducted in LMIC settings and selected non-LMIC studies only where the population, gene-drug pair or implementation environment was assessed directly transferable to LMIC populations or health systems.

### Information sources and search strategy

A full PubMed and Scopus search was conducted for publications published from 1 January 2020 to 31 December 2025. Keywords and database-appropriate subject terms related to cardiovascular disease, pharmacogenomics, precision medicine, and LMICs were used in the search strategy. Synonyms were linked via Boolean operators. We adjusted search strings with a medical librarian. We also manually screened the reference lists of relevant reviews and included articles to identify additional studies. The complete electronic search strategies for all databases are provided in S1 Table.

### Selection process

All retrieved references were imported into a reference manager, and duplicates were removed automatically and manually. Two reviewers independently screened titles and abstracts for relevance according to the predefined eligibility criteria. For every excluded study, the reason(s) for exclusion were documented and are displayed in the S1 Data. Disagreements at any stage were resolved by discussion or, if necessary, by a third reviewer.

### Data extraction and data items

A structured data extraction form was developed and piloted to ensure consistent and reproducible collection of information across reviewers. Two independent reviewers extracted study identifiers (first author, publication year, country of origin), study design and sample size, participant demographics (age, sex, baseline eGFR and comorbidities), details of the intervention (genes and variants tested, type of genetic test or panel, timing of testing (e.g., pre-emptive vs reactive), cardiovascular drug(s) involved and how the genetic results guided therapy.) and comparator, length of follow-up and outcomes grouped clinical efficacy outcomes (with definitions, such as what constituted a cardiovascular event), medication safety outcomes, intermediate or biomarker outcomes (laboratory or process measures) and implementation or process outcomes. Effect measures like hazard ratios, risk ratios, odds ratios and mean differences with their 95% confidence intervals were recorded. Means and standard deviations were derived from medians and interquartile ranges when necessary, using the established Wan method [11]. Where a study did not report a prespecified outcome, effect estimate, or subgroup result, this was recorded as ‘not reported’ rather than imputed; per protocol, study authors were not contacted for missing information. Missing summary statistics (standard deviations) were derived from reported medians and interquartile ranges using the Wan et al. method [11]; no other data imputation was performed. The extracted study-level dataset used for this review is provided in S2 Data.

### Risk of bias assessment

Two reviewers independently assessed the methodological quality of each included study using standardized tools tailored to specific study designs. For randomized controlled trials, we utilized the Cochrane Risk of Bias 2 (RoB 2) tool, which evaluates domains including the randomization process, deviations from intended interventions, missing outcome data, measurement of the outcome, and selection of the reported result [12]. For genetic association and pharmacogenomic profiling studies, we applied the Quality of Genetic Association Studies (Q-Genie) tool [13]. During this assessment, we paid particular attention to PGx-specific biases, including the validation of genotyping platforms, appropriate control for population stratification, and testing for Hardy-Weinberg equilibrium.

Observational and cross-sectional studies were appraised using the Joanna Briggs Institute (JBI) Critical Appraisal Checklist for Analytical Cross-Sectional Studies and the JBI Critical Appraisal Checklist for Prevalence Studies [14,15]. Furthermore, pharmacoeconomic and decision-analytic models were evaluated using the Consensus on Health Economic Criteria (CHEC) extended checklist to ensure rigorous assessment of cost and consequence valuations [16]. Risk-of-bias judgments (e.g., low, some concerns, or high risk for RoB 2; poor, moderate, or good for Q-Genie) were systematically recorded, and any disagreements between reviewers were resolved through consensus discussion. These assessments directly informed our evaluation of the overall certainty of evidence.

### Synthesis methods

#### Approach to synthesis.

We anticipated substantial heterogeneity across studies in design, interventions and outcomes and therefore did not perform a meta-analysis. Instead, we conducted a narrative synthesis following the Synthesis Without Meta-analysis (SWiM) guidelines [17]. Eight pre-specified narrative syntheses were conducted following SWiM principles, addressing study quality (PRISMA 20A), direction and magnitude of findings (20B), sources of heterogeneity (20C), and sensitivity of conclusions to study quality (20D). Studies were grouped according to prespecified thematic categories and gene-drug pairs. In particular, we organized the results into subsections focusing on:

1. **Clinical outcome benefits** of PGx-guided therapy (e.g., whether genotype-guided dosing improved patient outcomes compared with standard care).
2. **Medication safety outcomes** (e.g., reduction in adverse drug reactions with PGx use).
3. **Intermediate and biomarker outcomes** (such as changes in platelet reactivity or dosing accuracy due to PGx).
4. **Implementation outcomes and barriers** (including feasibility, acceptability, turnaround time, cost and resource considerations).
5. **Equity considerations**, such as the extent of representation of diverse populations and any disparities in implementation or outcomes.

For each thematic category we qualitatively summarized and compared findings across studies, highlighting consistencies and differences. Comparative results are presented narratively and, where feasible, in summary tables. We had planned to perform meta-analyses using a random-effects model if subsets of studies were sufficiently homogeneous, but no such subsets were identified. Heterogeneity was therefore explored qualitatively by comparing study contexts and results.

### Addressing reporting bias

Formal statistical evaluation of publication bias, such as funnel plot asymmetry testing, was not feasible due to the substantial clinical and methodological heterogeneity that precluded quantitative meta-analysis. To prospectively mitigate reporting bias, our literature search strategy was designed to be comprehensive, explicitly incorporating grey literature databases, clinical trial registries, and manual cross-referencing of prior systematic reviews to identify relevant unpublished or difficult-to-find studies. During the qualitative synthesis, the risk of bias due to missing results was assessed at the synthesis level for each of the predefined domains. Reviewers qualitatively evaluated the likelihood that evidence was missing based on study design, sample size, and the nature of the outcomes, paying particular attention to small candidate-gene association analyses and localized implementation reports where null, non-significant, or unfavorable findings are historically at a higher risk of being underreported.

### Certainty of Evidence

We assessed the certainty of evidence for key outcome domains using the Grading of Recommendations Assessment, Development and Evaluation (GRADE) approach [18]. Two reviewers independently evaluated the body of evidence for the main clinical efficacy outcomes (e.g., MACE, thrombotic events, major bleeding, mortality) and safety outcomes of PGx-guided therapy, considering five factors: risk of bias, inconsistency of results, indirectness (applicability to our population and LMIC context), imprecision (sample size and confidence interval width) and potential publication bias. Each outcome’s evidence was initially considered high certainty (for randomized evidence) or low certainty (for observational evidence) and was then downgraded or upgraded based on the GRADE criteria. Because no quantitative syntheses were performed, the GRADE assessment was qualitative and based on the range and consistency of effects reported across studies. The certainty ratings (high, moderate, low or very low) and justifications are summarized in Summary of Findings tables, which present for each key outcome the range of effect estimates, the number and type of contributing studies, overall risk-of-bias ratings and resulting GRADE judgments. These assessments provide context for interpreting the review’s conclusions and recommendations.

## Results

### Study selection

The initial systematic search across the selected databases (PubMed and Scopus) yielded a total of 515 records. After the removal of 65 duplicate records, 450 unique titles and abstracts were screened for relevance. This initial screening phase excluded 368 records based on title and abstract evaluation. Subsequently, the full texts of the remaining 82 articles were sought for retrieval, of which 6 reports could not be retrieved. The remaining 76 full-text articles were rigorously assessed against the predefined inclusion criteria. Of these, 52 reports were excluded for specific reasons, primarily including the use of an incorrect intervention (n = 22), study populations outside of low- and middle-income country (LMIC) contexts (n = 21), studies focusing exclusively on analytical validity rather than clinical outcomes (n = 5), studies with No relevant outcomes reported (n = 2), and studies with purely economic outcomes (n = 2). Ultimately, 24 studies met all inclusion criteria and were included in the final qualitative synthesis. The study selection process is depicted in a PRISMA 2020 flow diagram summarizing the number of records identified, screened, retrieved, excluded and included (Fig 1).

**Fig 1:**
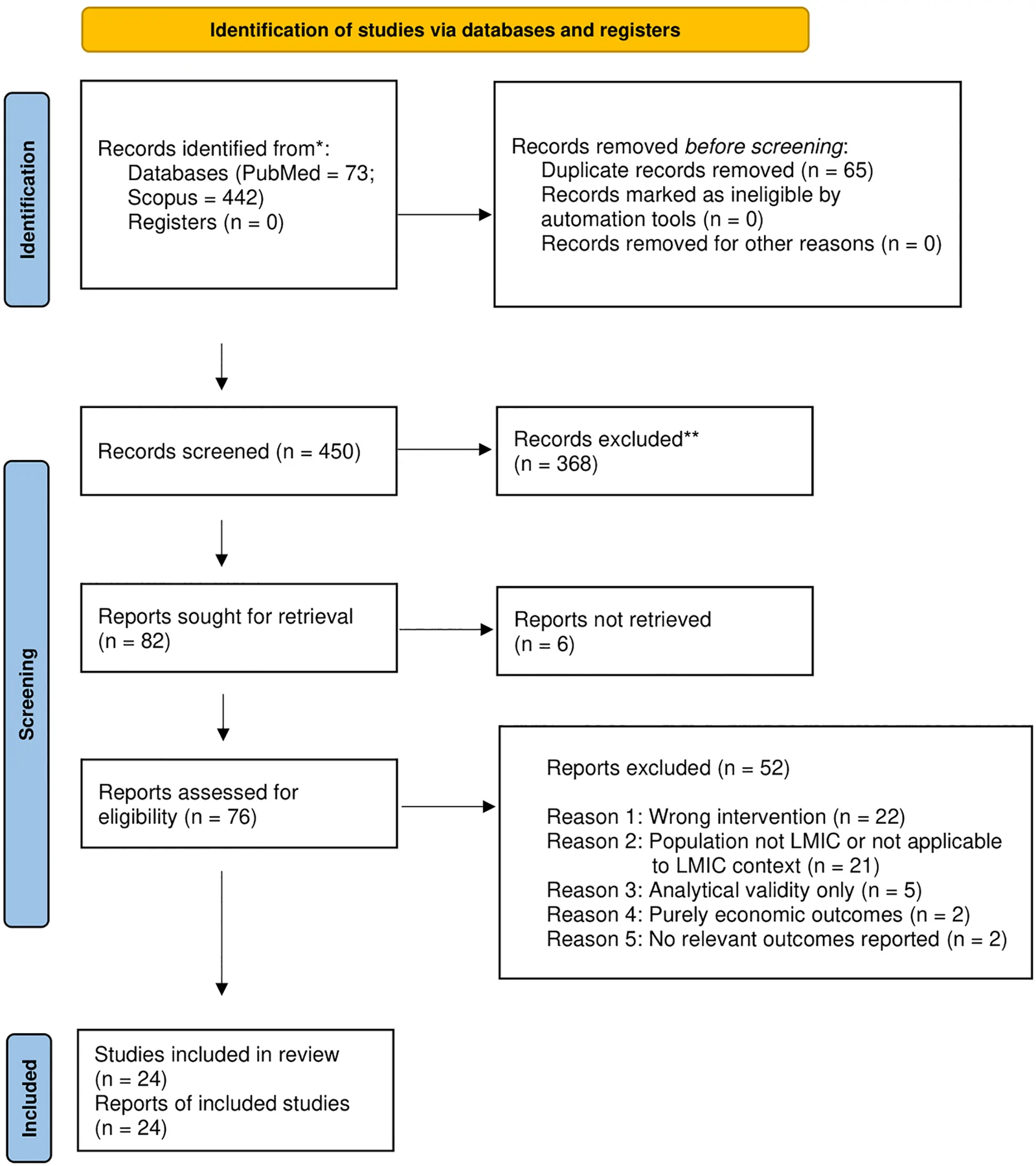
PRISMA 2020 flow diagram of study selection.

### Study characteristics

Twenty-four studies were included: one randomized controlled trial, nineteen observational/non-randomized studies, and four decision-analytic economic models. Sample sizes varied widely across study types, ranging from 82 participants in the smallest empirical clinical study to 527,500 participants in the largest EHR-linked biobank analysis. The most frequently evaluated gene–drug pair was CYP2C19 and antiplatelet therapy, particularly clopidogrel/ticagrelor in stroke or ischemic vascular disease [19–21], followed by warfarin pharmacogenomics involving CYP2C9, VKORC1, CYP4F2, CYP2C cluster variants, and related pharmacokinetic genes [22–28]. Antihypertensive pharmacogenomics focused on enalapril, amlodipine, hydrochlorothiazide, and resistant hypertension-related variants [29–33]. Additional studies evaluated statin-associated SLCO1B1 variants [34], empagliflozin response in heart failure [35], population pharmacogenomic diversity and ancestry-specific allele frequencies [36–38], and implementation, clinician/stakeholder perceptions, and health-system barriers to pharmacogenomics adoption [39–42]. Geographically, the evidence base spanned South Asia, East Asia, Southeast Asia, sub-Saharan Africa, the Middle East, Latin America and the Caribbean, and global multi-ancestry datasets.

Although this study was focused on low- and middle-income countries (LMICs), four studies from higher income Asian and Middle Eastern countries (Singapore, Malaysia, Qatar and UAE) were included in the final synthesis. These studies assessed several non-European populations with important genetic and infrastructural similarities to neighboring LMICs and hence provide highly translational evidence. In particular, they provide critical information on health economics, including cost-effectiveness of genotype-guided cardiovascular medicines, and practical knowledge on systemic implementation and stakeholder uptake. Retaining these studies was critical to strengthen the evidence basis for under-represented regional cohorts and to bridge the wider global equality gap in genetic medicine. The features of each included study are reported (Table 2).

**Table 2:** Individual study characteristics, comparisons, effects, safety notes of included studies (n = 24), grouped by gene-drug category.

Study ID	Country	Study Design	Population (N) & Baseline Status	Exposure/ Comparator	Follow-up Duration	Outcomes Assessed
**CYP2C19-guided antiplatelet/ clopidogrel therapy**						
Ranasinghe et al., 2025 [19]	Sri Lanka	Retrospective pharmacogenomic database analysis	690 Sri Lankans from anonymized exome-sequencing database; ethnicities: Sinhalese, Tamil, Moor, unknown	CYP2C19 and CES1 variant frequencies; compared with other global populations	N/A	Minor allele frequencies of CYP2C19 and CES1 variants relevant to clopidogrel metabolism
Narasimhalu et al., 2020 [20]	Singapore	Cost-effectiveness Markov model	Hypothetical cohort of 65-year-old Asian ischemic stroke patients	CYP2C19 genotype-guided therapy: ticagrelor for LOF carriers, clopidogrel for non-carriers vs universal clopidogrel	Lifetime model; yearly cycles to age 85	Costs, QALYs, ICER, ethnicity-specific cost-effectiveness
Cai et al., 2021 [21]	China	Decision tree + Markov cost-effectiveness model	Hypothetical acute minor stroke or high-risk TIA patients	CYP2C19 genotyping-guided antiplatelet therapy vs conventional clopidogrel + aspirin strategy	90-day decision tree plus 30-year Markov horizon	QALYs, costs, ICER, recurrent stroke, MI, extracranial hemorrhage, disability states
**Warfarin pharmacogenomics/ anticoagulation**						
Kaur et al., 2022 [22]	India	Prospective observational pharmacogenetic study	82 eligible analyzed from 240 screened; adults with AF and/or valve repair/replacement on stable warfarin anticoagulation	CYP2C9*2, CYP2C9*3, VKORC1 variants and clinical factors; no intervention comparator	Prospective study over 1 year	Stable weekly warfarin dose requirement; genetic and non-genetic predictors
Muyambo et al., 2022 [23]	South Africa and Zimbabwe	Observational real-world pharmacogenomic profiling study	503 patients on warfarin: 252 Black African, 251 Mixed Ancestry; common comorbidities included hypertension, heart failure, diabetes, arrhythmia, HIV	Genotyping of 73 variants in 29 pharmacogenes; ancestry and comedication profiling	N/A	PGx variant frequencies, interethnic differences, actionable variants, comedication-related gene-drug interaction relevance
Ndadza et al., 2021 [24]	South Africa and Zimbabwe	Cross-sectional pharmacogenetic association study	503 warfarin-treated participants: Black African and Mixed Ancestry groups	Warfarin pharmacokinetic gene variants by ancestry group; no clinical dosing comparator	Cross-sectional; no longitudinal follow-up	Warfarin maintenance dose associations with CYP2C cluster, CYP2C8, CYP2C9, CYP3A5, VKORC1, and other variants
Asiimwe et al., 2022 [25]	Uganda and South Africa	Genome-wide association study	548 Black African participants after QC; recruited from 12 outpatient sites; all on stable warfarin dose	Genome-wide SNP associations with warfarin/metabolite plasma concentrations	Cross-sectional sampling; no treatment follow-up reported	Plasma concentrations and ratios of warfarin enantiomers/metabolites; GWAS hits relevant to warfarin pharmacokinetics
Anand et al., 2024 [26]	India	Single-center open-label randomized controlled trial with blinded endpoint assessment; pharmacoeconomic model	168 new warfarin-requiring patients, 84 PGT-guided and 84 SOC; indications included AF, DVT, heart valve disease, mechanical/prosthetic valve, thromboembolism	Pharmacogenetic-guided warfarin dosing using CYP2C9*2, CYP2C9*3, VKORC1 vs standard-of-care warfarin dosing	3 months clinical follow-up; lifetime Markov model for AF economic analysis	Percent time in therapeutic INR range, time to therapeutic INR, adverse events, dose titrations, costs, QALYs, ICER
Hemati et al., 2024 [27]	Iran	Cost-effectiveness analysis with cross-sectional EQ-5D utility survey and decision tree model	105 MPHV patients surveyed for utilities; hypothetical cohort of 1,000 Iranian MPHV patients in model	PGx-guided warfarin dosing vs standard warfarin dosing	1-year model horizon	Utility scores, QALYs, costs, ICER, PTTR, bleeding and thrombotic event assumptions
Eljilany et al., 2023 [28]	Qatar	Cost-benefit decision-analytic model	Hypothetical cohort of warfarin-treated patients undergoing elective invasive procedure/surgery	Genotype-guided warfarin interruption algorithm vs standard interruption management	1-year decision-analytic follow-up	Benefit-cost ratio, adverse event states, bleeding, thromboembolism, death, procedure cancellation costs
**Antihypertensive pharmacogenomics/ resistant hypertension**						
Masilela et al., 2021 [29]	South Africa	Cross-sectional pharmacogenetic association study	284 Nguni adults with hypertension; on continuous antihypertensive therapy including enalapril; majority uncontrolled BP	SNPs in ABO, VEGFA, BDKRB2, NOS3, ADRB2 among enalapril-treated patients; controlled vs uncontrolled BP	Cross-sectional; no longitudinal follow-up	BP control response to enalapril; genotype/allele associations; gene-gene interactions
Masilela et al., 2022 [30]	South Africa	Cross-sectional pharmacogenetic association study	304 adults with hypertension on amlodipine; Swati, Xhosa, and Zulu groups; 52.3% uncontrolled hypertension	Thirteen amlodipine-associated SNPs; controlled vs uncontrolled BP	Cross-sectional; no longitudinal follow-up	BP control with amlodipine; SNP detection and genotype/allele associations
Katsukunya et al., 2024 [31]	South Africa	Retrospective matched case-control study	379 hypertensive patients: 190 resistant HTN cases, 189 non-resistant HTN controls; Black African and Mixed Ancestry	Genetic variants in ABCB1, ADRB1, CYP3A4, CYP3A5, NEDD4L, NR3C2; resistant vs non-resistant HTN	Retrospective records; no active follow-up	Resistant hypertension status; genotype and haplotype associations; CKD/LVH adverse outcome associations
Nyamupangedengu et al., 2025 [32]	South Africa	Targeted NGS pharmacogenetic association study	364 newly diagnosed, treatment-naive hypertensive adults with at least one follow-up; HCTZ monotherapy subset n = 103	Variants in 16 HCTZ response-related genes; heterogeneous treatment and HCTZ-only subgroup analyses	First follow-up targeted at 10–14 days, but many returned later; average 114 days	Variant discovery, BP control at follow-up, change in SBP/DBP, HCTZ monotherapy response
Katsukunya et al., 2025 [33]	South Africa	Retrospective age-, sex-, ethnicity-matched case-control study	379 hypertensive participants: 190 RHTN cases, 189 non-RHTN controls; African and Mixed Ancestry groups	CES1 and NOS3 variants/CNV; resistant vs non-resistant HTN	Retrospective records; no active follow-up	Resistant hypertension susceptibility; NOS3 and CES1 genotype/CNV associations; enalapril-related subgroup analysis
**Statin pharmacogenomics**						
Haldar et al., 2025 [34]	USA/UK-linked biobanks; African ancestry focus	Real-world EHR-linked biobank association study	527,500 participants in bilirubin analysis; secondary analysis included 578 severe statin-induced myotoxicity cases; Black and White participants analyzed	SLCO1B1 functional variants; variant carriers vs non-carriers; statin users vs matched controls for myotoxicity	Retrospective EHR-based; follow-up duration NR	Total bilirubin as OATP1B1 biomarker; severe statin-induced myotoxicity
**Heart failure/ SGLT2 inhibitor pharmacogenomics**						
Ain et al., 2025 [35]	Pakistan	Prospective pharmacogenetic association study	120 HFrEF patients initiated on empagliflozin; 99 genotyped; LVEF 25–45%, elevated BNP, stable GDMT ≥3 months	Empagliflozin 10 mg response by BDNF rs6265 and ATP2A2 rs1860561 genotype; no placebo/control arm	BNP and LVEF assessed at baseline, 6 months, and 12 months	EF response, BNP response; response defined as ≥5% EF increase or ≥20% BNP reduction
**Population pharmacogenomics/ ancestry and diversity**						
Runcharoen et al., 2021 [36]	Southeast Asia; controls from UAE and Greece	Multicounty targeted pharmacogene resequencing data report	1,571 DNA samples: Indonesia n = 562, Laos n = 100, Malaysia n = 105, Myanmar n = 100, Philippines n = 100, Thailand n = 100, Vietnam n = 100, UAE n = 100, Greece n = 304	100 pharmacogene resequencing across populations; SEA groups compared with UAE/Greece controls	N/A	Frequencies of PGx variants across 100 pharmacogenes; interpopulation variability; functional-impact variants
Sherman et al., 2024 [37]	Global 1000 Genomes populations	Population pharmacogenomic WGS analysis	2,504 unrelated 1000 Genomes samples from 26 global populations	PyPGx star-allele and structural-variant calling across 58 pharmacogenes	N/A	Star allele frequencies, predicted PGx phenotypes, structural variants, severe-consequence variants
Acosta-Monterrosa et al., 2025 [38]	Colombia	Cross-sectional ancestry-resolved PGx database analysis	4,462 PharmGKB annotations screened; 1,216 significant SNP annotations from 552 studies; Colombian populations: PLQ n = 34, CHG n = 96, ATQCES n = 404, ATQPGC n = 624, CLM n = 96	PharmGKB variant-drug annotations integrated with CÓDIGO allele frequencies; African- vs European-leaning Colombian populations	N/A	Ancestry-specific PGx allele-frequency differences, Spearman correlations, bibliometric evidence gaps
**PGx implementation, perceptions, barriers, and feasibility**						
Al-Mahayri et al., 2022 [39]	United Arab Emirates	Pilot PGx implementation study	160 adult CVD patients prescribed clopidogrel, warfarin, atorvastatin, or rosuvastatin	Real-time PCR genotyping of selected variants linked to clopidogrel, warfarin, and statins; no usual-care comparator	Baseline pilot report; follow-up planned for extended study but not reported	CYP2C19 metabolizer status, warfarin-relevant genotypes, SLCO1B1 statin-risk variant, PGx clinical recommendations
Che Mustapa et al., 2020 [40]	Malaysia	Cross-sectional stakeholder survey with PLS path modelling	421 adult respondents: 221 healthcare providers and 200 patients/family members	Predictors of intention to adopt PGx testing; no clinical comparator	N/A	Intention to adopt PGx; perceived benefits, risks, trust, engagement, religiosity, moral concerns
Kamp et al., 2022 [41]	South Africa	Cross-sectional clinician survey	109 practicing clinicians from four Johannesburg public academic hospitals	Clinician perceptions of PM-based CVD risk stratification tools; no comparator	Survey conducted Sept 2021–Mar 2022; no follow-up	Genetics/PM knowledge, perception, confidence, benefits, concerns, implementation barriers
Salas-Hernández et al., 2023 [42]	Latin America and Caribbean	Regional structured survey plus publication/trial landscape analysis	106 biomedical professionals from 17 countries; compared with 2014 survey n = 20	2022 LAC PGx implementation survey vs 2014 survey; perceived barriers and useful gene-drug pairs	N/A	PGx implementation barriers, usefulness of gene-drug pairs, publication/trial representation, changes since 2014

### Risk of bias in included studies

Overall, the evidence base showed mixed but generally acceptable methodological quality. The randomized study was judged as having “some concerns” overall, while economic evaluations met most CHEC criteria but commonly had limitations related to generalizability and ethical or distributional considerations. Genetic association studies ranged from poor to good quality, with most rated as moderate, mainly due to limitations in sample size/power, comparison-group definition, and testing of genetic assumptions. JBI-assessed studies also showed variable quality, with recurring uncertainty around sampling methods, measurement reliability, and control of confounding. These limitations were considered when interpreting the findings. A comprehensive breakdown of domain-level judgments for all included studies is provided in S1 File.

### Results of individual studies

Across the 24 included studies, pharmacogenomics-guided cardiovascular treatment showed a consistent directional advantage across all therapeutic domains evaluated. The greatest clinical evidence was for pharmacogenetically guided warfarin administration, which improved the time in therapeutic INR range approximately fivefold against conventional care (42.85% versus 8.8%; p < 0.00001) [26]. Clinical actionable population specific variants affecting response to antihypertensives [29–33], statins [34], and empagliflozin [35] were identified by genetic association studies, and allele frequency profiling confirmed the presence of high frequencies of clinically relevant variants in African and Asian ancestry populations, which are largely absent from current international guidelines [19,23–25,36–38]. Genotype-guided solutions were typically preferred in economic evaluations when testing costs were controlled [20,21,26–28]. Implementation studies regularly reported high clinical effectiveness and structural barriers of cost, workforce and absence of guidelines [39–42]. Full study-level outcome estimates by synthesis set are provided in S1 Appendix.

### Results of syntheses

The most robust conclusions were supported by convergent multi-design evidence: CYP2C9/VKORC1–warfarin pharmacogenomics (S2) and population-level actionable variant burden across LMIC-relevant populations (S7). Economic evidence for warfarin-guided therapy (S3) was context-sensitive and dependent on local genotyping costs, while single-study syntheses for statin myotoxicity (S5) and empagliflozin response (S6) remained hypothesis-generating. Implementation findings (S8) converged on a consistent barrier profile across three regions despite methodological heterogeneity. GRADE certainty was Moderate for anticoagulation quality only and Very Low for all remaining outcomes, reflecting the predominance of observational designs and the absence of directly measured hard cardiovascular endpoints. Complete synthesis narratives and sensitivity analyses are provided in the S2 Appendix.

### Assessment of risk of bias due to missing results

Overall, no clear evidence of selectively omitted results was identified, although reporting bias could not be excluded, particularly for small observational, candidate-gene, economic, implementation, and equity-focused studies where null or unfavorable findings may be underreported. The overall risk was judged low to moderate across syntheses, with no synthesis showing definitive evidence of high reporting bias (Table 3).

**Table 3:** Synthesis-level assessment of risk of bias due to missing results arising from reporting biases.

Synthesis assessed	Risk of bias due to missing results	Rationale
Genotype-guided antiplatelet therapy	Low to moderate	Main outcomes were generally reported, but publication of favorable CYP2C19-guided findings may be more likely.
Genotype-guided warfarin therapy	Low to moderate	Clinical, dosing, and economic outcomes were usually reported, although modelling assumptions and selective publication cannot be excluded.
Antihypertensive pharmacogenomics	Moderate	Many studies were small candidate-gene analyses; unpublished null associations are possible.
Statin pharmacogenomics	Moderate	Evidence was limited and focused on selected variants; ancestry-specific negative findings may be underreported.
Population pharmacogenomic diversity/ allele-frequency evidence	Low to moderate	Descriptive reporting was generally complete, but variant-panel selection may miss relevant unreported variants.
Implementation barriers, equity gaps, and pathways	Moderate	Barriers were reported narratively and heterogeneously; unsuccessful implementation experiences may be underrepresented.

### Certainty of the evidence

The overall certainty for all pre-specified outcomes ranged from very low to moderate. No outcome achieved high certainty. The highest level of assurance obtained, Moderate, only pertains to anticoagulation quality (time in therapeutic INR range [TTR]), which is supported by a significant amount of impact but is limited by open-label design flaws in the underlying data. We are quite uncertain about all the other outcomes including major adverse cardiovascular events (MACE), substantial haemorrhage and implementation results. This is because of the convergence of major methodological difficulties including reliance on observational studies, small sample sizes, surrogate endpoints and decision-analytic models rather than direct comparable measures. The full Summary of Findings (SoF) table is accessible in S2 Table.

## Discussion

This study synthesized data from 24 studies of pharmacogenomics (PGx)-guided cardiovascular therapy in low- and middle-income countries (LMICs) [19–42]. In all the therapeutic areas that were looked at, genotype-guided strategies consistently worked as well as or better than standard care. However, the main finding is that the biological promise is limited by structural inequity. It is challenging to implement this into practice because of infrastructure gaps, a lack of evidence for locally tailored guidelines, and the fact that LMIC populations are almost entirely left out of the global pharmacogenomic literature. We discuss these findings through five domains: clinical outcome benefits, medication safety outcomes, intermediate and biomarker outcomes, implementation outcomes and barriers, and equity considerations. We present an implementation framework, situate our findings in the larger literature, and discuss limitations and implications.

### Clinical outcome benefits

The most robust clinical signal came from warfarin pharmacogenomics. The sole included RCT (Anand et al. [26]) demonstrated approximately five-fold advantage in time in therapeutic INR range with pharmacogenetic-guided dosing (Fig 2).

**Fig 2:**
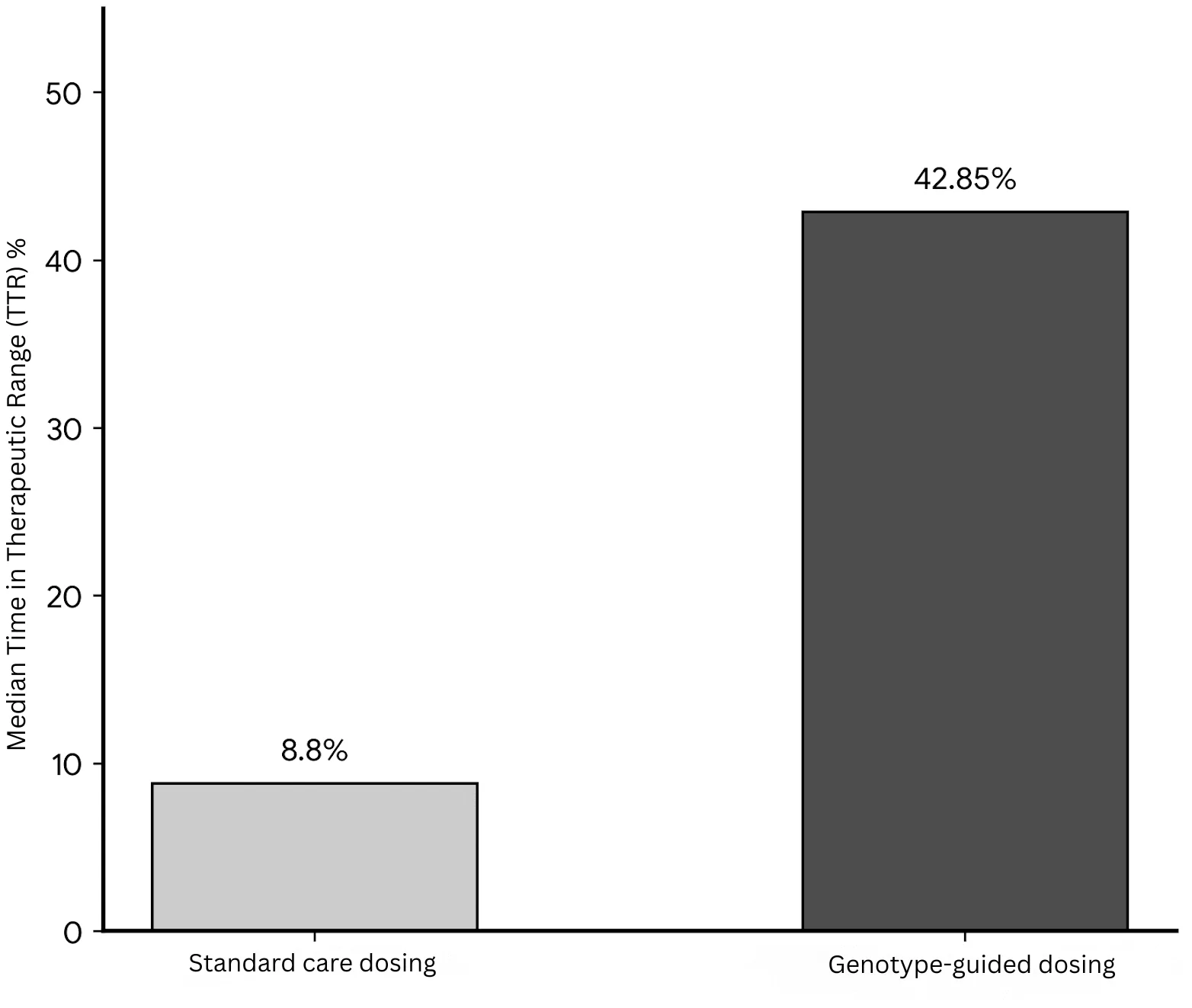
Impact of pharmacogenetic-guided dosing on time in therapeutic range. Bar chart showing median time in therapeutic range (TTR) for standard care versus genotype-guided dosing arms in a randomized controlled trial conducted in India. Genotype-guided dosing based on CYP2C9*2, CYP2C9*3, and VKORC1 variants which achieved a median TTR of 42.85% compared with 8.8% under standard care (p < 0.00001) over three months of follow-up. TTR, time in therapeutic range. Source: Anand et al. [26].

It also showed a 16-day reduction in time to reach therapeutic INR (Fig 3).

**Fig 3:**
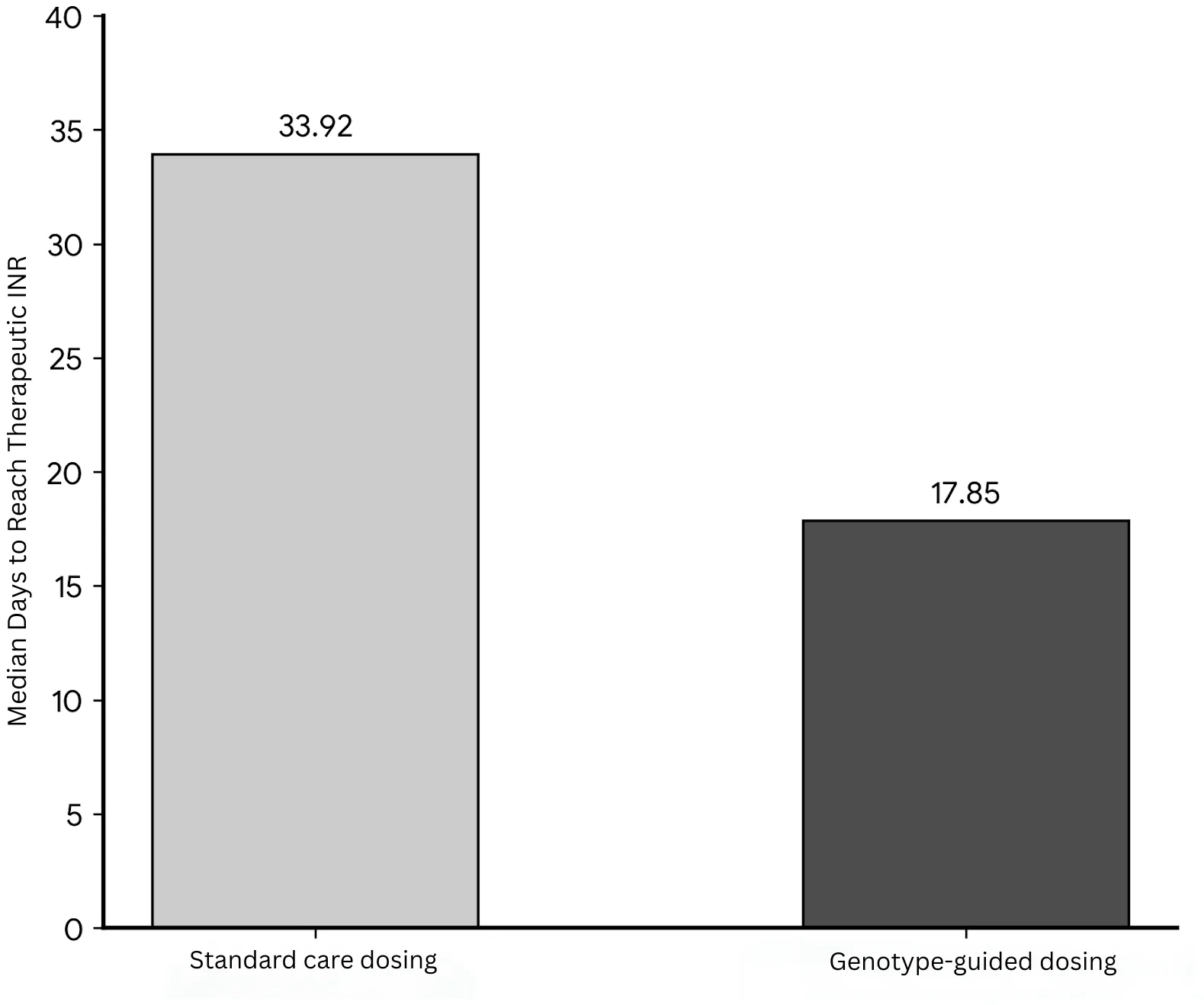
Time to reach therapeutic INR by dosing strategy. Bar chart showing median days to achieve therapeutic INR under standard care dosing (33.92 days) versus pharmacogenetic-guided warfarin dosing (17.85 days), representing a significant 16-day reduction (p = 0.002). Genotyping was performed for CYP2C9*2, CYP2C9*3, and VKORC1 variants in newly warfarin-requiring patients. INR, international normalized ratio; TRT, time to reach therapeutic INR. Source: Anand et al. [26].

This benefit is likely to be strengthened, rather than diminished, in LMIC settings as at least 31% of co-prescribed drugs in a Southern African warfarin cohort were contraindicated for warfarin therapy [23], CYP2C9*3 alone accounted for 29% of dose variability in a North Indian population [22], and a sub-Saharan African genome-wide association study validated ancestry-specific warfarin-response variants that are entirely absent from European-derived dosing algorithms [25]. Together, these results suggest that the individuals most at risk of harm from genotype-agnostic warfarin administration are also the populations least represented in the algorithms that now drive it.

For antiplatelet therapy, decision-analytic models from Singapore QALY [20] and China [21] found CYP2C19-guided clopidogrel prescribing to be cost-effective, supported by a pooled CYP2C19 loss-of-function allele prevalence of approximately 61% across the Chinese (65%), Indian (60%), and Malay (53%) populations sampled [20]. Cost-effectiveness at this scale in populations where loss-of-function alleles affect the majority of patients strengthens the case for genotype-guided antiplatelet selection as a default rather than an exception in these settings.

### Medication safety outcomes

Genetic variation predicted adverse drug reactions that are relevant to normal cardiovascular prescribing. SLCO1B1 rs59502379 was linked to a significantly higher risk of severe statin-induced myotoxicity in an African-ancestry cohort [34], which is consistent with SLCO1B1’s documented function in statin pharmacokinetics elsewhere but has not previously been measured at this scale in this ancestry group. Pre-prescription SLCO1B1 screening prior to high-intensity statin therapy may thus be a reasonably low-cost safety measure in areas where this variant is prevalent.

### Intermediate and biomarker outcomes

Three more relationships suggest to mechanisms that may inform future therapeutic decision-making, but none have been verified against hard clinical goals. A NEDD4L rs4149601 A/A genotype was related with a nearly four-fold greater risk of resistant hypertension [31], implying a genetic contribution to antihypertensive non-response that, if repeated, could assist identify individuals who require earlier escalation to combination medication. Separately, in a heart-failure cohort in Pakistan, a BDNF rs6265 CC genotype was linked with seven-fold increased odds of natriuretic-peptide (BNP) response and an approximately six-fold greater odds of ejection fraction (EF) response to empagliflozin (35). In the same cohort, the ATP2A2 rs1860561 GG genotype was associated with EF response, but with a lesser effect than BDNF, but had no association with BNP response. Before they may be used in practice, these biomarker signals must be validated against clinical heart failure outcomes.

### Implementation outcomes and barriers

Despite this pattern of therapeutic benefit, implementation readiness is much behind the underlying data. GRADE confidence was moderate for anticoagulation-quality outcomes but very low for all other outcomes evaluated, indicating the field’s near-complete dependence on observational methods and the lack of major adverse cardiovascular event (MACE) data. Despite generally positive attitudes toward precision-medicine approaches, surveyed clinicians in South Africa had a low mean PGx knowledge score (78%), with high testing costs and limited access to genetics services identified as the primary implementation barriers [41], and professionals surveyed across 17 Latin American and Caribbean countries rated the absence of local clinical guidelines as their single greatest barrier [42]. These findings are consistent with what the implementation-focused studies in this review have described throughout: infrastructural, workforce, and guideline deficiencies that limit translation no matter how good the underlying clinical evidence is.

### Equity considerations

These findings have significant implications for equity. African-ancestry people made up only 0.46% of the global pharmacogenomic literature compiled by Acosta-Monterrosa et al. [38], despite having some of the biggest ancestry-specific variances in drug-metabolizing enzyme activity reported in our research. DPYD intermediate-metabolizer phenotype frequencies alone vary by up to 69.8-fold across populations [37], though this figure should be interpreted cautiously, as the source study notes that part of this divergence may reflect differing “intermediate” versus “likely intermediate” phenotype-labeling conventions across reference databases rather than a purely biological difference. This underrepresentation is not a minor restriction of the evidence base; it is the primary equity issue that this review seeks to address: the people with the most differentiated, clinically actionable pharmacogenomic profiles have the fewest data available to act on. In a regional stakeholder-adoption survey, respondents’ self-reported religiosity (their degree of religious belief and practice) independently predicted their intention to adopt PGx testing [40]. Successful implementation strategies in LMICs will need to engage attitudinal and cultural determinants of adoption, not just technical and economic ones.

### A four-tier implementation framework: integrating evidence into a proposed model

To translate this evidence into an actionable strategy, we propose a four-tier, health-system-stratified framework for PGx implementation in LMICs, structured across clinical application, infrastructure, informatics, and human resources at each tier, with bidirectional flows of samples, results, and policy connecting them (Fig 4).

**Fig 4:**
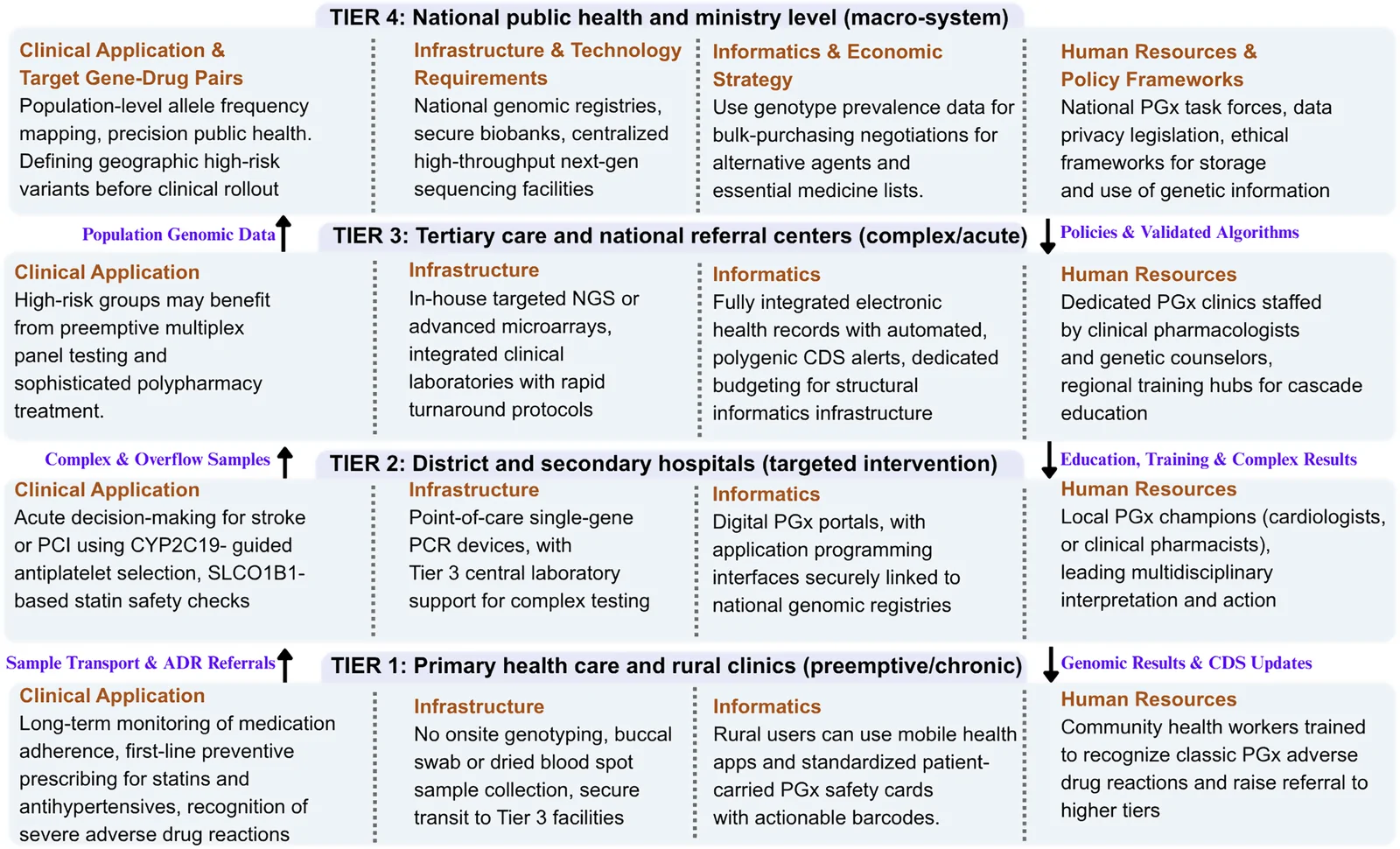
Tiered integration model for cardiovascular pharmacogenomics implementation in LMICs. **(A)** Tier 1 (primary and rural care) is designed to respond to the workforce and laboratory-capacity constraints documented in the implementation studies [39–42]. Buccal-swab DNA collection was used in two included South African studies [29,30], supporting minimally invasive sample collection with referral of specimens for off-site testing. Dried-blood-spot collection is proposed as an additional feasibility-based option where it has been locally validated; it was not evaluated in the included studies. Mobile-health applications and patient-carried PGx safety cards are likewise proposed mechanisms for preserving and communicating actionable results across fragmented care pathways. **(B)** Tier 2 (district hospitals), point-of-care, single-gene PCR testing would enable the CYP2C19-guided antiplatelet and SLCO1B1-based statin safety decisions demonstrated in this review [20,21,34], led by local PGx champions. **(C)** Tier 3 (tertiary and national referral centers) centralizes multiplex next-generation sequencing and automated clinical decision support [39]; In the economic evaluations examined here, this type of integration is associated with high cost-effectiveness. **(D)** Tier 4 (national/ministry level) governs population-level allele-frequency mapping, national genomic registries, bulk-purchasing negotiations, and data-privacy frameworks, a macro-system governance layer that oversees and coordinates Tiers 1-3 below it. Given the ancestry-representation gap discussed above under Equity Considerations, locally calibrated allele-frequency mapping at Tier 4 is not a discretionary refinement but a patient-safety requirement.

There are three main strategic points in this model.

**Strategic Point 1:** Sustainable PGx requires top-down national commitment and avoids imported algorithm misalignment. Tier 4 Enables all of them.

**Strategic Point 2:** Tertiary centers are the diagnostic and educational engines for complex, multivariable problems (e.g., warfarin dosing with changing renal function) and support Tiers 1–2.

**Strategic Point 3:** Most ongoing care occurs in district/primary care. Therefore, mHealth, durable result storage and patient-carried PGx safety records are central implementation mechanisms at Tiers 1–2

### Contextualization with existing literature

The barriers to PGx in LMICs are qualitatively different than implementation issues in HICs. The dominating friction in HIC research is physician resistance, reimbursement inconsistency, and EHR integration, while our findings reveal the absence of infrastructure, guideline vacuum, and personnel deficits functioning at the level of health system capacity. This is illustrated by the evidence for warfarin: African-specific variants (CYP2C9*8, *11; rs12777823 [24,25]) that are genomically confirmed but absent from international algorithms indicate that standard European-derived dosing would consistently lead to adverse outcomes in LMIC patients which PGx is intended to prevent. The economic statistics provide another LMIC-specific dimension, because cost-effectiveness is threshold sensitive, with Hemati et al. [27] establishing that PGx-guided warfarin dosing in Iran was only cost-effective when the cost of the test was below US$118, thus directly justifying the Tier 4 bulk-purchasing mechanism. That religiosity independently predicted PGx adoption intention [40] further highlights the deeply rooted character of LMIC implementation issues not reflected in any HIC frameworks.

### Limitations

The evidence base is hampered by the almost complete lack of RCT evidence: the only included RCT [26] was open-label, single-center (n = 168), and assessed at “some concerns” risk of bias due to significant performance bias risk, uncertain allocation concealment, and probable result selection. None of the examined studies quantified MACE, mortality or hospitalization directly, resulting in a Very Low GRADE certainty for the primary outcome. Genetic association studies were diverse in quality (Q-Genie scores 24–48), often failing to justify sample size, correction for multiple comparisons and confidence intervals, as in Ain et al. [35], where ORs of 7.70 and 5.97 were reported without precision data. Across all syntheses, there is a possibility of cohort overlap between two case-control studies from South Africa [31,33] leading to analytical reliance and moderate risk of publication bias. The review was confined to PubMed and Scopus, English language and the search window 2020–2025, which may have excluded foundational pre-2020 LMIC PGx investigations. Inclusion of four higher-income settings (Singapore, Malaysia, Qatar, UAE) adds marginal population indirectness, and lack of meta-analysis limits precision-weighted synthesis.

### Implications for practice, policy, and future research

For clinicians, the most immediately actionable priorities are CYP2C19-guided antiplatelet selection and pharmacogenetic warfarin dosing. By default, genotype-agnostic clopidogrel prescribing is clinically suboptimal given that a statistical majority of Asian stroke patients carry loss-of-function CYP2C19 alleles [20]. Association data suggest PGx recommendation for resistant hypertension and SLCO1B1 safety evaluation prior to high-intensity statin use in patients of African ancestry for antihypertensives and statins respectively, awaiting prospective validation. The evidence points to the following priorities for policymakers: (i) development of LMIC-specific, ancestry-informed PGx clinical guidelines, capitalizing on local frequency data from Southern African GWAS [25] and the SEAPharm network [36]; (ii) public funding of genotyping infrastructure, supported by the benefit-to-cost ratio of 4.0 [28] and the 96.94% simulation dominance of CYP2C19-guided therapy [21], leveraging national bulk-purchasing to drive test costs below WTP thresholds; and (iii) proactive enactment of genomic data privacy and sovereignty regulations prior to the establishment of national registries. The most urgent requirement for researchers is sufficiently powered, multi-site, blinded RCTs with MACE as the primary endpoint in geographically diverse LMIC populations followed by hybrid effectiveness-implementation trials to confirm the proposed four-tier framework. Considering that Africanancestry participants accounted for only 0.46% of global PGx study participants [38], the continued application of HIC-derived algorithms to LMIC patients represents an ongoing patient safety risk. Equity-centered population genomics programs for Africa, South Asia, and LAC are not optional complements to this agenda.

## Conclusion

This systematic review finds that PGx-guided cardiovascular therapy is biologically promising, directionally consistent, and economically justifiable in LMIC settings yet structurally limited by infrastructure deficits, guideline absence, and genomic inequity that global frameworks haven’t sufficiently addressed. The four-tier implementation model proposed here provides an evidence-grounded blueprint for equitable precision medicine across the full LMIC health system spectrum. Closing the gap between genomic knowledge and genomic justice will require both adequately powered LMIC-centered clinical trials and the coordinated policy action to build the systems that can deliver their findings to patients.

## Supporting information

S1 PRISMA Checklist PRISMA 2020 checklist. Completed Preferred Reporting Items for Systematic Reviews and Meta-Analyses checklist for this review.
(PDF)

S1 Protocol PROSPERO-registered review protocol. Protocol for this systematic review registered with PROSPERO (CRD420251150702).
(PDF)

S1 Table Search strategies. Complete PubMed and Scopus search strings, search dates, and screening search details used for study identification.
(DOCX)

S2 Table GRADE certainty of evidence assessment. Complete Summary of Findings (SoF) tables, along with a detailed, domain-by-domain GRADE appraisal.
(DOCX)

S1 Data Excluded studies with reasons. Dataset detailing the studies excluded during the full-text screening phase alongside their specific reasons for exclusion.
(XLSX)

S2 Data Extracted study-level data for included studies. Structured dataset containing study characteristics, interventions, outcomes, effect estimates, and study-level notes used in the synthesis.
(XLSX)

S1 File Risk-of-bias assessments. Detailed domain-level risk-of-bias and methodological quality appraisals (RoB 2, CHEC, JBI, Q-Genie) for all included studies.
(ZIP)

S1 Appendix Results of individual studies. Detailed study-level results, including outcome-specific estimates and supporting data.
(DOCX)

S2 Appendix Results of syntheses. Detailed synthesis-level results, including outcome-specific estimates and data organized by SWiM analysis sets.
(DOCX)
