# Supplementary material for: Pharmacogenomics-guided cardiovascular therapy in low- and middle-income countries: A systematic review of barriers, equity gaps, and pathways to personalized medicine
Source: PLOS Glob Public Health. 2026 Nov 4;6(11):e0006591. doi: 10.1371/journal.pgph.0006591 (PMC13734450; doi:10.1371/journal.pgph.0006591)
Supplement: Supplementary file 2 [file pgph.0006591.s002.pdf]

NIHR | National Institute for Health and Care Research

PROSPERO
International prospective register of systematic reviews

Pharmacogenomics Guided Cardiovascular Therapy in Low and Middle Income Countries: A Systematic Review of Barriers, Equity Gaps, and Pathways to Personalized Medicine

Md. Farid Reza Mashfi, Sumiya Sharmin Mou, Monalisa Riya, Nasiba Binte Bahar, Tasnuha Islam, Fayek Rafi Ahmad, Fabliha Fatima, Md. Elias Al-Mamun

Citation
Md. Farid Reza Mashfi, Sumiya Sharmin Mou, Monalisa Riya, Nasiba Binte Bahar, Tasnuha Islam, Fayek Rafi Ahmad, Fabliha Fatima, Md. Elias Al-Mamun. Pharmacogenomics Guided Cardiovascular Therapy in Low and Middle Income Countries: A Systematic Review of Barriers, Equity Gaps, and Pathways to Personalized Medicine. PROSPERO 2025 CRD420251150702. Available from https://www.crd.york.ac.uk/PROSPERO/view/CRD420251150702.

REVIEW TITLE AND BASIC DETAILS

Review title

Pharmacogenomics Guided Cardiovascular Therapy in Low and Middle Income Countries: A Systematic Review of Barriers, Equity Gaps, and Pathways to Personalized Medicine

Condition or domain being studied

Cardiovascular Therapy; Personalized medicine; Low Middle Income; Clinical Outcome; Implementation strategies

The purpose of this systematic review is to assess the effectiveness of cardiovascular therapy guided by pharmacogenomics in low- and middle-income (LMIC) nations. In order to guide therapy, minimize adverse drug responses, and enhance clinical outcomes, genetic testing (e.g., CYP2C19, CYP2D6, NAT2, and HLA alleles) is one of the personalized/precision medicine approaches that are applied to cardiovascular disorders. Additionally, it will evaluate the paths, equity gaps, and obstacles to pharmacogenomics implementation in LMIC healthcare systems.

Rationale for the review

Pharmacogenomics (PGx) presents a chance to enhance cardiovascular therapy through the reduction of adverse medication reactions, optimization of dosage, and facilitation of individualized treatment. While populations in low- and middle-income countries (LMICs) continue to be underrepresented while bearing a disproportionate burden of cardiovascular disease and a higher prevalence of some actionable genetic variations, the majority of PGx research originates from high-income nations. In addition to restricting clinical application, this disparity perpetuates unequal access to precision medicine.

The implementation of PGx is further hampered by a number of factors in LMIC health systems, such as inadequate laboratory infrastructure, exorbitant testing expenses, a lack of supportive regulations, and deficiencies in provider education. In these situations, the evidence is dispersed and frequently not combined to guide implementation. Thus, it is appropriate to conduct a comprehensive review on PGx-guided cardiovascular therapy in LMICs.

By consolidating available data, identifying barriers, mapping equity gaps, and outlining pathways to adoption, this review will provide a practical evidence base to guide policymakers, researchers, and

clinicians in advancing equitable precision medicine.

This review will offer a useful body of information to help clinicians, researchers, and policymakers advance equitable precision medicine by combining existing data, identifying obstacles, mapping equity gaps, and describing adoption routes.

Review objectives

1. Determine whether pharmacogenomic (PGx) testing or genotype-guided prescribing for cardiovascular drugs improves clinical outcomes in low- and middle-income countries (LMICs).

2. Identify barriers, equity gaps, and feasible pathways for implementing PGxguided cardiovascular therapy in LMIC health systems.

Keywords

Pharmacogenomics; Cardiovascular; Personalized medicine; Developing countries; Disparities; Implementation

Country

Bangladesh

ELIGIBILITY CRITERIA

Population

Included

Adolescents and adults receiving cardiovascular pharmacotherapy in LMICs (inpatient, outpatient, community). Studies involving patients with cardiovascular diseases (e.g., coronary artery disease, stroke, heart failure, atrial fibrillation) in LMIC settings or studies in other settings that yield insights applicable to LMIC populations (such as high allele frequency populations or addressing global diversity).

Excluded
We did not include multi-country or high-income country studies if their findings are not highly relevant to PGx implementation (e.g., major RCTs or meta-analyses of key gene–drug pairs)

Intervention(s) or exposure(s)

Included
Cardiovascular Therapy; Personalized medicine; Gene Therapy; Clopidogrel; Warfarin; Statins; Beta Blocking Agent

Comparator(s) or control(s)

Included
PICO tags selected: Usual Care; Complementary and alternative therapy

Study design 1 change

Only randomized study types will be included.

Included
• Randomized Controlled Trial / Controlled Clinical Trial / Pragmatic Clinical Trial

• Clinical Trial, Phase II–IV

• Adaptive Clinical Trial

• Equivalence Trial

• Observational Study

• Comparative Study

• Multicenter Study (if comparative)

• Evaluation Study

• Validation Study

Context

In this review, the function of cardiovascular therapy guided by pharmacogenomics will be investigated in low- and middle-income (LMIC) nations. In LMICs, where health systems have fewer resources and a higher incidence of adverse medication responses, cardiovascular illnesses are a leading cause of morbidity and mortality. The majority of existing research and recommendations are based on high-income populations, which leaves a genomic equity gap even though pharmacogenomics (PGx) has the potential to increase drug safety and efficacy. Therefore, research conducted in LMICs (2020–2025) that provide PGx allele frequencies, gene–drug correlations, clinical outcomes, or implementation findings will be the main emphasis of this review. Excluded studies will be those conducted in high-income environments.

TIMELINE OF THE REVIEW

Date of first submission to PROSPERO

17 October 2025

Review timeline 1 change

Review start date: 19 September 2025. Review end date: 10 May 2026.

Date of registration in PROSPERO

18 October 2025

AVAILABILITY OF FULL PROTOCOL

Availability of full protocol

A full protocol has not been written.

SEARCHING AND SCREENING

Search for unpublished studies

Only published studies will be sought.

Main sources that will be searched

The main databases to be searched are *PubMed* and *Scopus*.

Search language restrictions

The review will only include studies published in English.

Search date restrictions

Databases will be searched for articles published from 1 January 2020 and before by 31 December 2025.

Other methods of identifying studies

No other methods will be used.

Link to search strategy 1 change

A full search strategy has been uploaded to PROSPERO. The PDF may be accessed through this link https://www.crd.york.ac.uk/PROSPEROFILES/d809061540e87853450a727addcc8a29.pdf.

Selection process

Studies will be screened independently by at least two people (or person/machine combination) with a process to resolve differences.

Other relevant information about searching and screening

None

DATA COLLECTION PROCESS

Data extraction from published articles and reports

Data will be extracted independently by at least two people (or person/machine combination) with a process to resolve differences.

Authors will not be contacted for further information.

Study risk of bias or quality assessment 1 change

Risk of bias will be assessed using: *Cochrane RoB-2*

CHEC Checklist, JBI Checklist, Q-Genie

Data will be assessed independently by at least two people (or person/machine combination) with a process to resolve differences.

Additional information will be sought from study investigators if required information is unclear or unavailable in the study publications/reports.

Reporting bias assessment 1 change

Risk of bias from missing results is qualitatively assessed at the synthesis level due to heterogeneity preventing funnel plots. Reviewers consider study design, sample size, and outcome when estimating the probability of missing evidence, paying special attention to small or localised studies that underreport null findings.

Certainty assessment 1 change

The review will use the GRADE (Grading of Recommendations Assessment, Development, and Evaluation) approach to assess the certainty of evidence for each critical outcome, classifying it as high, moderate, low, or very low.

OUTCOMES TO BE ANALYSED

Main outcomes

The primary outcome is to evaluate the effect of pharmacogenomics-guided cardiovascular therapy on major adverse cardiovascular events (MACE) among adults in low- and middle-income countries.

Additional outcomes

Secondary outcomes include hospitalization/readmission, major bleeding and other adverse drug reactions, all-cause and cardiovascular mortality, genotyping turnaround time, prescriber changes (dosage/switches), intermediate measures (platelet reactivity, INR time-in-therapeutic-range, LDL-C change), and implementation metrics (adoption, acceptability, feasibility, fidelity, equity, and costs).

PLANNED DATA SYNTHESIS

Strategy for data synthesis 1 change

Due to substantial clinical, methodological, population, and outcome heterogeneity across the included studies, a statistical meta-analysis will not be performed. Instead, data will be combined using a formal narrative synthesis in accordance with the Synthesis Without Meta-Analysis (SWiM) reporting guidelines.

Studies will be stratified into pre-specified analysis sets according to the specific gene-drug pair (e.g., CYP2C19-clopidogrel, CYP2C9/VKORC1-warfarin) or the implementation domain. Within each synthesis group, the data will be systematically evaluated and combined by:

1. Summarizing the study design, sample size, geographic setting, and methodological quality (appraised via design-appropriate tools such as RoB 2, CHEC, Q-Genie, or JBI checklists).

2. Reporting the direction, magnitude, precision, and consistency of the findings without statistical aggregation. Adjusted estimates and hard clinical or decision-analytic endpoints will be prioritized over unadjusted or descriptive results.

3. Qualitatively assessing the sources of heterogeneity across dimensions such as population ancestry, clinical indication, genotyping platform, study design, and health-system context.

4. Performing qualitative sensitivity analyses to evaluate whether directional conclusions remain robust when studies of lower quality, smaller sample sizes, or those utilizing surrogate outcomes are excluded.

Finally, the overall certainty of the synthesized evidence for key outcomes will be assessed using the Grading of Recommendations Assessment, Development and Evaluation (GRADE) approach, appropriately adapted for narrative synthesis without pooled effect estimates.

CURRENT REVIEW STAGE

Stage of the review at this submission 1 change

Review stage	Started	Completed
Pilot work	✓	✓
Formal searching/study identification	✓	✓
Screening search results against inclusion criteria	✓	✓
Data extraction or receipt of IPD	✓	✓
Risk of bias/quality assessment	✓	✓
Data synthesis	✓	✓

Review status

The review is currently planned or ongoing.

Publication of review results

Results of the review will be published in English.

REVIEW AFFILIATION, FUNDING AND PEER REVIEW

Review team members

Md. Farid Reza Mashfi. ORCID: 0009-0008-3445-1260. University of Asia Pacific. Bangladesh.

No conflict of interest declared.

Sumiya Sharmin Mou (review guarantor and contact) University of Asia Pacific. Bangladesh.

No conflict of interest declared.

Monalisa Riya. University of Asia Pacific. Bangladesh.

No conflict of interest declared.

Nasiba Binte Bahar. University of Dhaka. Bangladesh.

No conflict of interest declared.

Tasnuha Islam. University of Asia Pacific. Bangladesh.

No conflict of interest declared.

Fayek Rafi Ahmad. University of Asia Pacific. Bangladesh.

No conflict of interest declared.

Fabliha Fatima. University of Asia Pacific. Bangladesh.

No conflict of interest declared.

Dr Md. Elias Al-Mamun. University of Dhaka. Bangladesh.

No conflict of interest declared.

Named contact

Sumiya Sharmin Mou (ssmou@uap-bd.edu). University of Asia Pacific. Bangladesh.

Review affiliation

University of Asia Pacific

Funding source

Review has no funding and no agreed support from an academic institution and is done in authors' own time.

Peer review

There has been no peer review of this planned review.

ADDITIONAL INFORMATION

Review conflict of interest

Declared individual interests are recorded under team member details.. No additional interests are recorded for this review.

Medical Subject Headings

Pharmacogenetics; Genetic Testing; Cardiovascular Diseases; Precision Medicine; Developing Countries; Alleles; Warfarin; Clopidogrel; Cytochrome P-450 CYP2C19; Cytochrome P-450 CYP2D6; Delivery of Health Care; Humans

Revision note 1 change

This revision updates the protocol to accurately reflect our finalized methodology, including extending the review end date to May 10, 2026. To appropriately appraise the diverse study designs included, the risk of bias assessment tools were expanded beyond RoB-2 to include the

CHEC Checklist, JBI Checklists, and Q-Genie. Furthermore, we updated the protocol to specify that reporting bias is qualitatively assessed and the GRADE approach is utilized to evaluate the certainty of findings. Finally, the data synthesis strategy (SWiM) was clarified in a better way.

SIMILAR REVIEWS

Check for similar records already in PROSPERO

PROSPERO identified a number of existing PROSPERO records that were similar to this one (last check made on 18 September 2025). These are shown below along with the reasons given by that the review team for the reviews being different and/or proceeding.

- Algorithmic Bias and Equity in AI-Powered ECG Interpretation for Low- and Middle-Income Countries [published 4 June 2025] [CRD420251040561]. The review was judged **not to be similar**
- Implementation of Cardiovascular Imaging AI in Low and Middle Income Countries: A Systematic Review [published 17 January 2025] [CRD42025634337]. The review was judged **not to be similar**
- Effects of household air pollution (HAP) on cardiovascular diseases in low and middle income countries: a systematic review and meta-analysis. [published 10 May 2021] [CRD42021248800]. The review was judged **not to be similar**

PROSPERO version history 1 change

- Version 2.0, published 07 May 2026
- Version 1.0, published 18 Oct 2025

Disclaimer

The content of this record displays the information provided by the review team. PROSPERO does not peer review registration records or endorse their content.

PROSPERO accepts and posts the information provided in good faith; responsibility for record content rests with the review team. The guarantor for this record has affirmed that the information provided is truthful and that they understand that deliberate provision of inaccurate information may be construed as scientific misconduct.

PROSPERO does not accept any liability for the content provided in this record or for its use. Readers use the information provided in this record at their own risk.

Any enquiries about the record should be referred to the named review contact
