# Supplementary material for: Pharmacogenomics-guided cardiovascular therapy in low- and middle-income countries: A systematic review of barriers, equity gaps, and pathways to personalized medicine
Source: PLOS Glob Public Health. 2026 Nov 4;6(11):e0006591. doi: 10.1371/journal.pgph.0006591 (PMC13734450; doi:10.1371/journal.pgph.0006591)
Supplement: Supplementary file 3 [file pgph.0006591.s003.docx]

**Search Strategy**

**1. Core effectiveness in LMICs:**

| **Database (Platform)** | **Search Date** | **Search Strategy (Full Boolean String)** | **Limits Applied** | **Results Retrieved** |
| --- | --- | --- | --- | --- |
| **PubMed (MEDLINE)** | 31-12-2025 | ("Pharmacogenetics"[Mesh] OR "Pharmacogenomics" OR pharmacogenomic*[tiab] OR pharmacogenetic*[tiab] OR genotype-guided[tiab] OR "genetic testing"[tiab]) AND ("Cardiovascular Diseases"[Mesh] OR cardiovascular[tiab] OR coronary[tiab] OR "myocardial infarction"[tiab] OR "acute coronary syndrome"[tiab] OR "heart failure"[tiab] OR hypertens*[tiab] OR clopidogrel[tiab] OR "P2Y12"[tiab] OR warfarin[tiab] OR statin*[tiab] OR simvastatin[tiab] OR "beta-blocker*"[tiab] OR anticoagulant*[tiab] OR CYP2C19[tiab] OR CYP2C9[tiab] OR VKORC1[tiab] OR SLCO1B1[tiab] OR CYP2D6[tiab] OR HLA[tiab] OR NAT2[tiab]) AND ("Developing Countries"[Mesh] OR "low income countr*"[tiab] OR "middle income countr*"[tiab] OR LMIC[tiab] OR Africa[tiab] OR Asia[tiab] OR "Latin America"[tiab]) | **Time frame**:2020-2025  **Study designs:**   - Randomized Controlled Trial / Controlled Clinical Trial / Pragmatic Clinical Trial - Clinical Trial, Phase II–IV - Adaptive Clinical Trial - Equivalence Trial - Observational Study - Comparative Study - Multicenter Study (if comparative) - Evaluation Study - Validation Study | 7 |
| **Scopus (Elsevier)** | 31-12-2025 | TITLE-ABS-KEY(("pharmacogenomic*" OR "pharmacogenetic*" OR "genotype-guided" OR "genetic testing") AND (cardiovascular OR coronary OR "myocardial infarction" OR "acute coronary syndrome" OR "heart failure" OR hypertens* OR clopidogrel OR "P2Y12" OR warfarin OR statin* OR simvastatin OR "beta-blocker*" OR anticoagulant* OR CYP2C19 OR CYP2C9 OR VKORC1 OR SLCO1B1 OR CYP2D6 OR HLA OR NAT2) AND ("developing countr*" OR "low income countr*" OR "middle income countr*" OR LMIC OR Africa OR Asia OR "Latin America")) | **Time frame**:2020-2025  **Document Type:** Article | 119 |

**2. Implementation, equity, and barriers in LMICs:**

| **Database (Platform)** | **Search Date** | **Search Strategy (Full Boolean String)** | **Limits Applied** | **Results Retrieved*** |
| --- | --- | --- | --- | --- |
| **PubMed (MEDLINE)** | 31-12-2025 | ("Pharmacogenetics"[Mesh] OR "Pharmacogenomics" OR pharmacogenomic*[tiab] OR pharmacogenetic*[tiab] OR genotype-guided[tiab] OR "genetic testing"[tiab]) AND (implementation[tiab] OR barrier*[tiab] OR challenge*[tiab] OR adoption[tiab] OR uptake[tiab] OR feasibility[tiab] OR acceptability[tiab] OR "health system*"[tiab] OR policy[tiab] OR "clinical practice"[tiab] OR "routine care"[tiab] OR "precision public health"[tiab]) AND ("Developing Countries"[Mesh] OR "low income countr*"[tiab] OR "middle income countr*"[tiab] OR LMIC[tiab] OR Africa[tiab] OR Asia[tiab] OR "Latin America"[tiab]) | **Time frame**:2020-2025  **Study designs:**   - Randomized Controlled Trial / Controlled Clinical Trial / Pragmatic Clinical Trial - Clinical Trial, Phase II–IV - Adaptive Clinical Trial - Equivalence Trial - Observational Study - Comparative Study - Multicenter Study (if comparative) - Evaluation Study - Validation Study | 13 |
| **Scopus (Elsevier)** | 31-12-2025 | TITLE-ABS-KEY(("pharmacogenomic*" OR "pharmacogenetic*") AND (implementation OR barrier* OR challenge* OR adoption OR uptake OR feasibility OR acceptability OR "health system*" OR policy OR "clinical practice" OR "routine care" OR "precision public health") AND ("developing countr*" OR "low income countr*" OR "middle income countr*" OR LMIC OR Africa OR Asia OR "Latin America")) | **Time frame**:2020-2025  **Document Type:** Article | 78 |

**3. Cost-effectiveness of PGx-guided therapy:**

| **Database (Platform)** | **Search Date** | **Search Strategy (Full Boolean String)** | **Limits Applied** | **Results Retrieved*** |
| --- | --- | --- | --- | --- |
| **PubMed (MEDLINE)** | 31-12-2025 | ("Pharmacogenetics"[Mesh] OR "Pharmacogenomics" OR pharmacogenomic*[tiab] OR pharmacogenetic*[tiab] OR genotype-guided[tiab] OR "genetic testing"[tiab]) AND ("Costs and Cost Analysis"[Mesh] OR cost-effective*[tiab] OR "economic evaluation"[tiab] OR "cost utility"[tiab] OR "cost benefit"[tiab] OR "health economic*"[tiab]) | **Time frame**:2020-2025  **Study designs:**   - Randomized Controlled Trial / Controlled Clinical Trial / Pragmatic Clinical Trial - Clinical Trial, Phase II–IV - Adaptive Clinical Trial - Equivalence Trial - Observational Study - Comparative Study - Multicenter Study (if comparative) - Evaluation Study - Validation Study | 55 |
| **Scopus (Elsevier)** | 31-12-2025 | TITLE-ABS-KEY(("pharmacogenomic*" OR "pharmacogenetic*") AND (cost-effective* OR "economic evaluation" OR "cost utility" OR "cost benefit" OR "health economic*")) | **Time frame**:2020-2025  **Document Type:** Article | 245 |
