# Supplementary material for: Pharmacogenomics-guided cardiovascular therapy in low- and middle-income countries: A systematic review of barriers, equity gaps, and pathways to personalized medicine
Source: PLOS Glob Public Health. 2026 Nov 4;6(11):e0006591. doi: 10.1371/journal.pgph.0006591 (PMC13734450; doi:10.1371/journal.pgph.0006591)
Supplement: Supplementary file 4 [file pgph.0006591.s004.docx]

# **Table A. Summary of Findings (SoF) GRADE Certainty of Evidence Assessment**

PGx = pharmacogenomics; MACE = major adverse cardiovascular events; TTR = time in therapeutic range; TRT = time to reach therapeutic INR; ADR = adverse drug reaction; RCT = randomized controlled trial; ICER = incremental cost-effectiveness ratio; QALY = quality-adjusted life year; ICUR = incremental cost-utility ratio; WTP = willingness-to-pay; LAC = Latin America and the Caribbean; LMICs = low- and middle-income countries. Certainty ratings: ⊕⊕⊕⊕ High | ⊕⊕⊕◯ Moderate | ⊕⊕◯◯ Low | ⊕◯◯◯ Very Low. Domain shading: green = Not serious; amber = Serious; red = Very serious. ↓ = one-level downgrade; ↓↓ = two-level downgrade.

| **Certainty assessment** | | | | | | | **No. of patients** | | **Effect** | | **Certainty** | **Importance** |
| --- | --- | --- | --- | --- | --- | --- | --- | --- | --- | --- | --- | --- |
| **No. of studies** | **Study design** | **Risk of bias** | **Inconsistency** | **Indirectness** | **Imprecision** | **Other considerations** | **PGx-guided** | **Standard care** | **Relative (95% CI)** | **Absolute effect / Key finding** |  |  |
| **Major Adverse Cardiovascular Events (MACE); Primary outcome** | | | | | | | | | | | | |
| **0** empirical; **4** models [20,21,27,28] | Decision-analytic Markov / decision-tree models | very serious | not serious | very serious | serious | Moderate concern (publication bias | Modelled | Modelled | — | **Not directly measured.** MACE not reported as an empirical endpoint in any included study. All evidence is model-projected (event rates sourced from CHANCE, BRIDGE, and published registries). | **⨁◯◯◯ Very Low** | **CRITICAL** |
| **Anticoagulation Quality: Time in Therapeutic INR Range (TTR) and Time to Reach Therapeutic INR (TRT); Secondary outcome (warfarin)** | | | | | | | | | | | | |
| **4** studies n=1,401 [22,24,25,26] | 1 RCT [26] + 1 prospective observational [22] + 1 GWAS [25] + 1 cross-sectional [24] | serious | not serious | not serious | serious | Low concern | **84** (RCT-PGx arm); 503–548 (observational, no control arm) | **84** (RCT-SOC arm); (observational, no comparator) | (Narrative synthesis; no meta-analysis) | **RCT [26]:** TTR 42.85% vs. 8.8% (p<0.00001); **TRT:** 17.85 vs. 33.92 days (p=0.002) **Supporting:** CYP2C9*3+VKORC1+BMI explain 47.5% dose variance [22]; CYP2C9*8 GWAS p=9.53×10⁻¹² [25] | **⨁⨁⨁◯ Moderate** | **CRITICAL** |
| **Major Bleeding and Other Adverse Drug Reactions (ADRs); Secondary outcome** | | | | | | | | | | | | |
| **1** RCT [26] n=168 + genetic data [19,23,34] | 1 RCT + genetic association / biobank studies | serious | not serious | serious | very serious | Moderate concern (publication bias) | 84 | 84 | — | **RCT [26]:** CRNMB 1.08% vs. 1.12% (p=0.976); Grade 3/4 thrombosis 2.16% vs. 3.36% (p=0.683); **0 major bleeds** in either arm. SLCO1B1 rs59502379: OR=2.85 (1.08–7.52) for statin myotoxicity [34] | **⨁◯◯◯ Very Low** | **CRITICAL** |
| **All-cause and Cardiovascular Mortality; Secondary outcome** | | | | | | | | | | | | |
| **0** empirical; 4 models [20,21,27,28] | Decision-analytic models (mortality as model parameter) | very serious | not serious | very serious | very serious | Moderate concern | Modelled | Modelled | — | **Not directly measured.** ICH mortality assumed 48.6%; ischemic stroke mortality 11.7% [27]; Eljilany [28]: 6% increase in adverse-event-free health state with PGx warfarin interruption (no mortality estimate) | **⨁◯◯◯ Very Low** | **CRITICAL** |
| **Hospitalization / Readmission; Secondary outcome** | | | | | | | | | | | | |
| **0** empirical; 4 models [20,21,27,28] | Decision-analytic models (hospitalization absorbed as cost input) | very serious | not serious | very serious | very serious | Moderate concern | Modelled | Modelled | — | **Not directly measured.** Hospitalization not reported as a comparative endpoint in any empirical study; absorbed as cost input in economic model structures only. | **⨁◯◯◯ Very Low** | **IMPORTANT** |
| **Genotyping Turnaround Time; Secondary outcome (implementation process metric)** | | | | | | | | | | | | |
| **0** formal studies [26,39,41] | Qualitative mention only; no comparative measurement | very serious | not serious | very serious | very serious | Moderate concern | N/A | N/A | — | **Not quantitatively measured.** Anand et al. [26]: testing preceded initiation (duration NR); Al-Mahayri et al. [39]: described as 'on-demand' without quantified time; Kamp et al. [41]: qualitative reference only. | **⨁◯◯◯ Very Low** | **IMPORTANT** |
| **Prescriber Changes (Dosage Adjustments / Drug Switches) ;Secondary outcome** | | | | | | | | | | | | |
| **1** RCT [26] n=168; 1 pilot [39] n=160; observational [22–25] | 1 RCT + 1 pilot (no comparator) + observational studies | serious | not serious | serious | very serious | Moderate concern | **84** (RCT); 160 (pilot) | **84** (RCT); ;(pilot: no comparator) | — | **RCT [26]:** Median dose titrations 5 (PGx) vs. 8 (SOC), p=0.001 **Pilot [39]:** 96% of patients received ≥1 PGx recommendation; 46.9% clopidogrel-avoidance recommended; 80% warfarin dose-guidance issued; 31.5% SLCO1B1 statin-risk alert generated. No prescribing adherence follow-up in any study. | **⨁◯◯◯ Very Low** | **IMPORTANT** |
| **Intermediate Biological Process Measures (Platelet Reactivity; BP Control; Statin Myotoxicity; LDL-C Change; Empagliflozin EF / BNP Response); Secondary outcome** | | | | | | | | | | | | |
| **8** observational / biobank studies [23,29–35] | Cross-sectional association + retrospective case-control + EHR-linked biobank | serious | not serious | serious | serious | Moderate concern (candidate-gene selective reporting) | 82–527,500 (range across studies) | Carrier vs. non-carrier (no intervention comparator) | **OR range:** 0.13–7.70 (across gene–drug pairs) | **BP control [29–33]:** NEDD4L rs4149601 OR=3.82 (1.67–9.07); NOS3 rs3918188 aOR=0.13 (0.04–0.41); ACE rs4291 aOR=0.23 (0.06–0.85) **Statin myotoxicity [34]:** SLCO1B1 rs59502379 OR=2.85 (1.08–7.52) **SGLT2i response [35]:** BDNF rs6265 CC OR=7.70^g^; ATP2A2 rs1860561 GG OR=5.97 (EF); CIs not reported **Warfarin PK [23,24,25]:** CYP2C9*8 GWAS p=9.53×10⁻¹²; CYP2C9*3 explains 29% dose variance (R²=0.29) | **⨁◯◯◯ Very Low** | **IMPORTANT** |
| **Implementation Metrics (Adoption, Acceptability, Feasibility, Fidelity, Equity, and Costs); Secondary outcome** | | | | | | | | | | | | |
| **4** surveys [39–42]; 4 models + 1 RCT [20,21,26–28] | 1 pilot + 3 cross-sectional surveys + 4 decision-analytic economic models + 1 RCT (economic sub-analysis) | serious | not serious | serious | serious | Moderate concern (positive implementation bias) | 106–1,571 (surveys); Modelled (economic analyses) | N/A (surveys); Modelled (economic analyses) | — | **Cost-effectiveness:** ICER CNY 13,553/QALY [21]; S$33,839/QALY [20]; ₹35,962/QALY [26]; BCR 4.0 [28]; ICER US$2,474/QALY – not cost-effective at Iranian threshold [27] **Barriers [41,42]:** Cost 89%; personnel shortage 83%; low knowledge 78%; guidelines rated 9.06/10 dominant barrier across 17 LAC countries **Acceptability [40]:** Perceived benefits β=0.371 (p<0.001); trust β=0.312 (p<0.001)^h^ **Feasibility [39]:** 96% clinical recommendation yield; high actionability confirmed in resource-variable LMIC-adjacent setting | **⨁◯◯◯ Very Low** | **IMPORTANT** |
