# Supplementary material for: Pharmacogenomics-guided cardiovascular therapy in low- and middle-income countries: A systematic review of barriers, equity gaps, and pathways to personalized medicine
Source: PLOS Glob Public Health. 2026 Nov 4;6(11):e0006591. doi: 10.1371/journal.pgph.0006591 (PMC13734450; doi:10.1371/journal.pgph.0006591)
Supplement: Supplementary file 6 [file pgph.0006591.s008.docx]

## Results of Individual Studies

Across the 24 included studies, investigators used diverse designs including descriptive pharmacogenomic (PGx) profiling, case control association and genome‑wide association studies (GWAS), a single randomized controlled trial, pharmacoeconomic models and mixed‑methods implementation or stakeholder surveys to explore precision medicine in cardiovascular and thrombotic conditions.

Most effective clinical difference was for warfarin in a pharmacogenetic experiment by Anand et al. [26]. In this study PGx-guided dosing increased per-protocol time in therapeutic INR range approximately fivefold (42.85% vs 8.8%, p<0.00001) and reduced time to attain therapeutic INR (17.85 vs 33.92 days; p=0.002 per the trial's abstract, p=0.006 per the trial's results table) compared to standard treatment. Ain et al. [35] reported the largest odds ratios in pharmacogenetic association studies in patients with heart-failure treated with empagliflozin. The BDNF rs6265 CC genotype was associated with a seven-fold greater odds of BNP response (OR=7.70; 95% CI 2.4–24.65; p<0.001) and an almost six-fold greater odds of EF response (OR=5.97; 95% CI 2.04–17.46; p=0.001) relative to CT/TT carriers. The ATP2A2 rs1860561 GG genotype was associated with EF response (OR=0.09; 95% CI 0.01–0.97; p=0.048) but showed no association with BNP response (OR=0.14; 95% CI 0.01–1.7; p=0.128); note that the source paper's own abstract states an EF odds ratio of 5.97 for ATP2A2, which does not match its Table 4 or Discussion (both reporting OR=0.09) and appears to be a transcription error in the source publication. Katsukunya et al. [31] observed that, in resistant hypertension, the CYP3A5*3 CC genotype was protective (OR=0.44; 95% CI 0.22–0.89), while those with the NEDD4L rs4149601 A/A genotype had approximately four-fold greater odds of resistant hypertension (OR=3.82; 95% CI 1.67–9.07) and those with ADRB1 rs1801252 G/G three-fold greater odds. Recent multi-ancestry studies of statin adverse events have also implicated rare transporter variants. Haldar et al. [34] associated carriers of SLCO1B1 rs59502379 with increased risk of severe statin-induced myotoxicity (OR=2.85; 95% CI 1.08–7.52) and noted that population-specific haplotypes beyond common rs4149056 may impact safety.

The PGx trials on warfarin highlighted the variability of variation effects across populations. Kaur et al. [22] constructed a dosage-prediction model in North India using CYP2C9*3, VKORC1 (rs9923231) and body-mass index, explaining over half of inter-individual dose variability. In a related Southern African cohort of 503 patients on warfarin, Muyambo et al. [23] found that at least 31% of co-prescribed drugs were contraindicated for warfarin therapy, underscoring the polypharmacy risk in this setting. Ndadza et al. [24] found that CYP2C9*8, *11 and CYP2C rs12777823 variations were related to lower maintenance dosages in Black Africans, while CYP2C9*2, CYP2C8*4 and VKORC1 −1639G>A were significant in a mixed-ancestry population. Asiimwe et al. [25] replicated relationships for CYP2C9*8, *9, *11 and rs12777823, and identified rs11188082 as the top dose-response signal (p=1.5×10−17) in a sub-Saharan Black African GWAS. In addition to warfarin dose, population-wide surveys of allele frequencies showed geographical differences: A Sri Lankan cohort of 690 participants revealed a minor-allele frequency of CYP2C19 rs12769205 and rs4244285 of 42% by Ranasinghe et al. [19], much higher than most reference groups.

Economic evaluations tended to support genotype‑guided solutions, if the cost of testing was minimal. Hemati et al. [27] observed that PGx-guided warfarin dose increased quality of life (EQ-5D utility 0.76 vs. 0.68; p<0.0001) but the incremental cost-effectiveness ratio (ICER) was US$2,474 per QALY beyond the local willingness-to-pay barrier until genotyping costs reduced below roughly US$118. Cai et al. [21] found that CYP2C19 genotyping for acute minor stroke or transient ischaemic attack is highly cost-effective in China (ICER =CNY 13,553/=US$1,931 per QALY) and Narasimhalu et al. [20] projected an ICER of S$33,839/QALY for genotype-guided antiplatelet therapy in Singapore, below a S$60,000 threshold. Eljilany et al. [28] calculated a benefit-to-cost ratio of 4.0 and per-patient cost reduction of roughly US$574 for genotype-guided warfarin interruption, but Anand et al. [26] reported an ICUR of INR 35,962/QALY with a 90 % chance of cost-effectiveness at willingness-to-pay of INR 71,630.

Implementation and stakeholder studies also highlighted readiness and barriers in the actual world. In a UAE pilot, Al‑Mahayri et al. [39] found that approximately 47% of adults would need alternatives to clopidogrel and 96% received at least one PGx recommendation, indicating the high actionability of PGx results. A survey of South African clinicians by Kamp et al. [41] indicated that 78% had limited PGx expertise and 89% mentioned cost as a key barrier. Salas-Hernández et al. [42] indicated that a high perceived need for guidelines (score 9.06/10) was the main implementation hurdle in 17 countries of Latin America and the Caribbean. Che Mustapa et al. [40] found that perceived advantages (β=0.371), trust (β=0.312), engagement (β=0.272) and religiosity (β=0.133) predicted intention to use PGx testing, whereas perceived dangers were negatively linked.

Taken together, these findings illustrate that PGx applications in cardiovascular medicine are clinically promising, vary by ancestry and setting, and may be economically attractive when testing costs are controlled; however, effective implementation will require targeted education, clear guidelines and stakeholder engagement. Individual study results are summarized in Tables A–G below according to the SWiM analysis sets.

### Table A: Antiplatelet Pharmacogenomics (CYP2C19–Clopidogrel / Ticagrelor)

| **Study (Author, Year)** | **Outcomes Evaluated** | **Group 1 Summary Statistics** | **Group 2 Summary Statistics** | **Effect Estimate (95% CI)** | **p-value** |
| --- | --- | --- | --- | --- | --- |
| Ranasinghe et al. (2025) [19] | Minor allele frequencies (MAFs) of CYP2C19 (rs12769205, rs4244285) and CES1 (rs71647871) variants | Sri Lankan cohort (n = 690) | European, African American, and East Asian reference populations | MAFs: rs12769205 = 42.1% (95% CI not reported); rs4244285 = 42.0%; CES1 rs71647871 = 0.2% | p < 0.05 for all CYP2C19 reference comparisons |
| Narasimhal et al. (2020) [20] | Cost-effectiveness (ICER) and QALYs in ischaemic stroke patients | CYP2C19-genotype-guided antiplatelet therapy (ticagrelor substitution for LOF-allele carriers) | Universal clopidogrel monotherapy | +0.27 QALYs; ICER = S$33,839/QALY (Chinese S$30,755; Indian S$33,177; Malay S$41,470) | Not explicitly reported; PSA confirmed cost-effectiveness in large majority of iterations |
| Cai et al. (2021) [21] | Cost-effectiveness (ICER) and QALYs in acute minor stroke/TIA patients | CYP2C19 genotyping to guide DAPT selection | Standard therapy (implicit base case) | +0.031 QALYs; ICER = CNY 13,552.74/QALY (US$1,930.59) | Not explicitly reported; PSA showed beneficial in 96.94% and cost-effective in 95.71% of simulations |

### Table B: Anticoagulation Pharmacogenomics (CYP2C9 / VKORC1 / CYP2C Cluster Warfarin)

| **Study (Author, Year)** | **Outcomes Evaluated** | **Group 1 Summary Statistics** | **Group 2 Summary Statistics** | **Effect Estimate (95% CI)** | **p-value** |
| --- | --- | --- | --- | --- | --- |
| Kaur et al. (2022) [22] | Predictors of stable warfarin dose variability | Stable warfarin therapy patients (n = 82 analysable) | Not applicable | CYP2C9*3 accounted for 29% of dose variability; VKORC1 −1639G>A contributed 18%; Total variance explained 47.5% | p < 0.001 for CYP2C9*3 |
| Muyambo et al. (2022) [23] | Pharmacogene allele frequencies (CYP2C9, CYP2C cluster); co-prescribed drug interaction risk | Black Africans (BA; n = 252) | Mixed Ancestry (MA; n = 251) | CYP2C9*2 MAF: 2% (BA) vs 8% (MA); CYP2C9*3 MAF: 0% (BA) vs 10% (MA); rs12777823 MAF: 49% (BA) vs 41% (MA); ≥31% of co-prescribed drugs contraindicated for warfarin therapy | Not explicitly reported |
| Ndadza et al. (2021) [24] | Association of SNPs with warfarin maintenance dose | Black Africans (BA; n = 252) | Mixed Ancestry (MA; n = 251) | BA dominant haplotype: CYP2C9*8 and rs12777823; MA frequent haplotypes: CYP2C9*2 and *3. VKORC1 associated in both | rs12777823 (BA): p < 0.05; VKORC1 (both): p < 0.001 |
| Asiimwe et al. (2023) [25] | Warfarin pharmacokinetics (plasma enantiomer/metabolite concentrations) | Sub-Saharan black-African patients (n = 548) | Not applicable | CYP2C9*8, *9, *11, and rs12777823 passed replication threshold; rs11188082 associated with S/R-warfarin ratio | Replication p < 3.21 × 10−4; GWAS top SNP p = 1.55 × 10−17 |
| Anand et al. (2024) [26] | Time in therapeutic range (TTR), time to reach therapeutic INR (TRT), ICUR | Pharmacogenetic-guided (PGT) arm (n = 84) | Standard-of-care (SOC) arm (n = 84) | Median TTR: 42.85% (95% CI 21.4–66.75) vs 8.8% (95% CI 0–27.2); TRT: 17.85 vs 33.92 days; ICUR: ₹35,962/QALY | TTR: p < 0.00001; TRT: p = 0.002 (abstract) / p = 0.006 (table) |
| Hemati et al. (2024) [27] | Cost-effectiveness (ICER) of PGx-guided vs standard dosing | PGx-guided warfarin dosing | Standard warfarin dosing | Base case: not cost-effective (ICER exceeded US$3,303.2/QALY threshold); PSA: cost-effective if test cost ≤US$118 | Not applicable |
| Eljilany et al. (2023) [28] | Cost-benefit analysis for pre-procedural warfarin interruption | Pharmacogenetic-guided algorithm | Standard of care (SOC) | Benefit-to-cost ratio = 4.0; Net benefit 6% improvement in no-adverse-event health state | Not applicable |

### Table C: Antihypertensive Pharmacogenomics (South Africa)

| **Study (Author, Year)** | **Outcomes Evaluated** | **Group 1 Summary Statistics** | **Group 2 Summary Statistics** | **Effect Estimate (95% CI)** | **p-value** |
| --- | --- | --- | --- | --- | --- |
| Masilela et al. (2021) [29] | SNP associations with controlled hypertension (enalapril response) | Controlled hypertension (BP <140/90 mmHg) | Uncontrolled hypertension (BP ≥140/90 mmHg) | ADRB2 rs1042714 GC: AOR 2.31 (1.02–5.23); BDKRB2 rs1799722 CT: AOR 2.74 (1.19–6.28); VEGFA rs699947 C: AOR 0.37 (0.15–0.94) | ADRB2 p=0.044; BDKRB2 p=0.017; VEGFA p=0.037; 3-way interaction p=0.0005 |
| Masilela et al. (2022) [30] | SNP associations with controlled hypertension (amlodipine response) | Controlled hypertension (BP < 140/90 mmHg) | Uncontrolled hypertension (BP ≥ 140/90 mmHg) | ACE rs4291 AA genotype associated with controlled hypertension: AOR = 0.23 (0.06–0.85) | ACE rs4291 AA: p = 0.028 |
| Katsukunya et al. (2024) [31] | SNP associations with resistant hypertension (RHTN) | Cases (RHTN; n = 190) | Controls (non-RHTN; n = 189) | CYP3A5*3 CC: OR 0.44 (0.22–0.89) [multivariable-adjusted]; CYP3A5 C–C–A haplotype: OR 0.73 (0.54–0.97) [unadjusted]; NEDD4L AA: OR 3.82 (1.67–9.07) [multivariable-adjusted] | CYP3A5*3 CC: p=0.02 [adjusted]; CYP3A5 C–C–A hap: p=0.03 [unadjusted]; NEDD4L AA: p=0.001 [adjusted] |
| Nyamupangedengu et al. (2025) [32] | Variant associations with HCTZ monotherapy response | HCTZ monotherapy sub-group (n = 103) | Not applicable | 46% achieved control; GNB3 rs1025450259 associated with BP change; 0 of 882 variants significant after correction | GNB3 change: p < 0.001 (corrected) |
| Katsukunya et al. (2025) [33] | NOS3/CES1 variants and CES1 CNV associations with RHTN | Cases (RHTN) | Controls (non-RHTN) | NOS3 rs3918188 A allele (Africans): OR 0.13 (0.04–0.41); CES1 haplotype (MA): OR 0.54 (0.37–0.78) | NOS3 A allele: p < 0.001; CES1 haplotype: p = 0.001 |

### Table D: Statin-Related Pharmacogenomics (SLCO1B1)

| **Study (Author, Year)** | **Outcomes Evaluated** | **Group 1 Summary Statistics** | **Group 2 Summary Statistics** | **Effect Estimate (95% CI)** | **p-value** |
| --- | --- | --- | --- | --- | --- |
| Haldar et al. (2025) [34] | Total bilirubin elevation and severe statin-induced myotoxicity | Black participants (1KG-AFR-like genetic similarity) | White participants | Bilirubin: rs77271279 beta=8.57; rs59502379 beta=6.85; rs4149056 beta=4.01. Myotoxicity: rs59502379 OR=2.85 (1.08–7.52); rs77271279 OR=1.75 (0.62–4.73) | Bilirubin: p=1.1×10−17, p=7.4×10−12, p=6.0×10−5 respectively |

### Table E: Heart Failure Pharmacogenomics (Empagliflozin / SGLT2i)

| **Study (Author, Year)** | **Outcomes Evaluated** | **Group 1 Summary Statistics** | **Group 2 Summary Statistics** | **Effect Estimate (95% CI)** | **p-value** |
| --- | --- | --- | --- | --- | --- |
| Ain et al. (2025) [35] | BNP response (≥20% reduction) and EF response (≥5% increase) | BNP responders | BNP non-responders | BDNF CC genotype: EF response OR=5.97 (95% CI 2.04–17.46); BNP response OR=7.70 (95% CI 2.4–24.65). ATP2A2 GG genotype: EF response OR=0.09 (95% CI 0.01–0.97); BNP response OR=0.14 (95% CI 0.01–1.7, not significant) | BDNF EF: p=0.001; BDNF BNP: p<0.001; ATP2A2 EF: p=0.048; ATP2A2 BNP: p=0.128 (ns) |

### Table F: Global Pharmacogenomic Profiling and Allele Frequency Mapping

| **Study (Author, Year)** | **Outcomes Evaluated** | **Group 1 Summary Statistics** | **Group 2 Summary Statistics** | **Effect Estimate (95% CI)** | **p-value** |
| --- | --- | --- | --- | --- | --- |
| Runcharoen et al. (2021) [36] | Pharmacogene variant frequencies (CYP2C19, CYP2D6, ABCB1, SLCO1B1) | Southeast Asian individuals (n = 1,267) | UAE and Greece reference populations | SLCO1B1 rs2291075 (T) MAF: 0.400 (Myanmar), 0.470 (Vietnam) | Not explicitly reported |
| Sherman et al. (2025) [37] | Star-allele and phenotype frequencies across 58 pharmacogenes | 1000 Genomes Project (n = 2,504; 5 super-populations) | PharmGKB reference frequencies | AFR frequencies for CYP2C9 differ markedly from EUR; 210 uncharacterised small nucleotide variants identified; DPYD intermediate-metabolizer phenotype frequency diverged 69.8-fold between 1KGP-AFR and PharmGKB-SSA reference data (the source study notes this may partly reflect a ‘likely intermediate’ vs ‘intermediate’ labeling difference across databases rather than a purely biological difference) | Not applicable |
| Acosta-Monterrosa et al. (2025) [38] | Annotation of allele frequencies across 4,462 PharmGKB variants | Colombian sub-populations (n = 1254; Palenque, Chocó, and 3 European-ancestry) | Global PharmGKB evidence base (n = 651,532) | Palenque vs European-ancestry populations similarity: r² = −0.20 to −0.02; 28 SNPs high in PLQ but low in EUR | Not applicable |

### Table G: Pharmacogenomics Implementation: Pilots, Attitudes, and Barriers

| **Study (Author, Year)** | **Outcomes Evaluated** | **Group 1 Summary Statistics** | **Group 2 Summary Statistics** | **Effect Estimate (95% CI)** | **p-value** |
| --- | --- | --- | --- | --- | --- |
| Al-Mahayri et al. (2022) [39] | Clinical PGx variant frequencies and implementation recommendations | UAE patients (n = 160) | gnomAD reference population | CYP2C19*2 = 17.4%; CYP2C19*3 = 17.95%; 96% received actionable recommendation | p < 0.01 vs gnomAD for CYP2C19*2 and *3 |
| Che Mustapa et al. (2020) [40] | Determinants of intention to adopt PGx testing | Malaysian healthcare providers and patients (n = 421) | Not applicable | Perceived benefits: β=0.371; Trust in key players: β=0.218; Engagement: β=0.201; Perceived risks: β=−0.096; Religiosity: β=0.133 | p < 0.001 (benefits, trust, engagement); p < 0.05 (risks); p < 0.01 (religiosity); p > 0.05 (moral concerns) |
| Kamp et al. (2022) [41] | Knowledge, perceptions, confidence, and barriers regarding PM-based CVD risk | South African clinicians (n = 109) | Not applicable | 76% (95% CI 67.0–83.7) had positive mean perception of PM-based approaches; low mean PGx knowledge score (78%); high testing cost and limited access to genetics services identified as key implementation barriers in the source abstract, though not separately quantified as percentages; most-cited concerns were potential adverse effects on patients’ lives/insurance (73%) and limited cross-population transferability of risk scores (53%) | Knowledge score improvement: p < 5×10−5; Practice association: p < 0.05 |
| Salas-Hernández et al. (2023) [42] | Barrier perception and gene/drug pair usefulness | LAC researchers and clinicians (n = 106) | Not applicable | Top barrier: Need for national clinical guidelines (9.06/10); Top pair: CYP2C19/clopidogrel (4.98/5) | Not applicable |
