# Supplementary material for: Pharmacogenomics-guided cardiovascular therapy in low- and middle-income countries: A systematic review of barriers, equity gaps, and pathways to personalized medicine
Source: PLOS Glob Public Health. 2026 Nov 4;6(11):e0006591. doi: 10.1371/journal.pgph.0006591 (PMC13734450; doi:10.1371/journal.pgph.0006591)
Supplement: Supplementary file 7 [file pgph.0006591.s009.docx]

# Results of Syntheses

Given the considerable heterogeneity across clinical, methodological, population and outcome domains, a statistical meta-analysis was not conducted; rather, a formal narrative synthesis was conducted following the Synthesis Without Meta-Analysis (SWiM) reporting criteria. Studies were grouped into eight pre-specified analysis sets by gene–drug pair or implementation domain: (S1) CYP2C19–clopidogrel and antiplatelet therapy; (S2) CYP2C9 and VKORC1–warfarin dose and anticoagulation quality; (S3) economic evaluations of pharmacogenomic-guided warfarin therapy; (S4) antihypertensive pharmacogenomics and resistant hypertension; (S5) SLCO1B1–statin-induced myotoxicity; (S6) BDNF and ATP2A2–empagliflozin response in heart failure with reduced ejection fraction (HFrEF); (S7) population pharmacogenomic profiling actionable variant burden across LMIC and LMIC-adjacent populations; and (S8) implementation, stakeholder barriers, and pharmacogenomics adoption. Four PRISMA 2020 elements were methodically addressed in each synthesis.

PRISMA Item 20A: Summaries of contributing studies, including design, sample size, geographic context, and quality-appraisal outcome The design was evaluated for quality using suitable tools: Cochrane Risk of Bias 2 (RoB 2) for the single RCT [26]; Consolidated Health Economic Evaluation Reporting Standards (CHEC) checklist for the four decision-analytic economic models [20,21,27,28]; Q-Genie for eleven genetic association studies [19,22–25,29–33,35]; and the Joanna Briggs Institute (JBI) prevalence and analytical cross-sectional checklists for the remaining eight studies [34,36–42]. For PRISMA Item 20B, direction, magnitude, precision, and consistency of findings were provided without aggregation; preference was given to adjusted estimates, clinical endpoints, and decision-analytic outcomes over descriptive or unadjusted data when available. We qualitatively evaluated reasons for variation across dimensions including ancestry, therapeutic indication, study design, genotyping platform, outcome definition, follow-up duration, economic model structure and health-system environment for PRISMA Item 20C. For PRISMA Item 20D, qualitative sensitivity analyses were performed to determine whether the directional conclusions were intact after removing studies of lesser quality, smaller sample size or surrogate outcome focus.

.

**Table A. Summary of results of syntheses without meta-analysis**

| **Synthesis**  **(Gene–Drug / Domain)** | **Contributing Evidence**  **(PRISMA 20A)** | **Direction & Key Finding**  **(PRISMA 20B)** | **Main Heterogeneity Sources**  **(PRISMA 20C)** | **Sensitivity / Robustness**  **(PRISMA 20D)** |
| --- | --- | --- | --- | --- |
| **S1. CYP2C19 – clopidogrel / antiplatelet therapy** | 3 studies: Ranasinghe 2024 [19] (allele-frequency; Q-Genie 24/66 Poor), Narasimhalu 2020 [20] (CHEC 15/19), Cai 2021 [21] (CHEC 17/19). | Supportive. LOF allele prevalence 42% (Sri Lanka), 61% (Singapore). ICER S$33,839/QALY (Singapore) and CNY 13,553/QALY (China); both below WTP. No direct clinical outcome RCT available. | Outcome type (frequency vs. ICER), population (Sri Lanka, Singapore, China), model structure, comparator strategy, WTP threshold. | Direction stable excluding Poor-quality Ranasinghe [19]; certainty rests primarily on two economic models. Generalizability gaps in CHEC items 17 and 19 for both models. |
| **S2. CYP2C9 / VKORC1 – warfarin dose and anticoagulation quality** | 4 studies: Kaur 2022 [22] (Q-Genie 36/66 Moderate), Ndadza 2021 [24] (29/66 Poor), Asiimwe 2022 [25] (48/66 Good), Anand 2024 [26] (RoB-2 'Some concerns'). | Supportive. CYP2C9/VKORC1 explained 47.5% of dose variability [22]; African-specific variants (CYP2C9*8, *11, rs12777823) drive dose in sub-Saharan populations [24,25]. RCT: TTR 42.85% vs. 8.8% (p<0.00001); TRT 17.85 vs. 33.92 days (p=0.006) [26]. | Ancestry (South Asian vs. sub-Saharan African), endpoint (dose / PK concentration / TTR), follow-up duration, genotyping platform, adjustment for non-genetic covariates. | Consistent direction without Ndadza [24]; removing the RCT [26] weakens clinical translation but not the pharmacogenomic-association signal. Conclusion robust across three quality tiers. |
| **S3. PGx-guided warfarin – economic evaluations** | 2 models: Hemati 2024 [27] (CHEC 15/19; 4 gaps); Eljilany 2023 [28] (CHEC 17/19; 2 gaps). Also, the economic component embedded within the RCT [26] (CHEC not separately assessed). | Mixed / context-sensitive. Ellijay [28]: BCR=4.0; cost reduction US$573.72/patient. Hemati [27]: EQ-5D 0.76 vs. 0.68 (p<0.0001); ICER not cost-effective at current test cost; cost-effective if testing ≤US$118. Anand [26]: ICUR ₹35,962/QALY; 90% probability cost-effective at WTP ₹71,630. | Decision context (dosing initiation vs. pre-procedural interruption), time horizon, cost structure, payer perspective, adverse-event probability assumptions, local WTP thresholds. | Excluding Hemati [27] overstates benefit; excluding Eljilany [28] removes positive CBA signal. Including Anand's RCT economic arm [26] strengthens LMIC cost-effectiveness evidence. Overall: conditionally favorable, highly context-sensitive. |
| **S4. Antihypertensive pharmacogenomics and resistant hypertension** | 5 South African observational studies: Masilela 2021 [29] (38/66 Moderate), Masilela 2022 [30] (37/66 Moderate), Katsukunya 2024 [31] (46/66 Good), Nyamupangedengu 2025 [32] (40/66 Moderate), Katsukunya 2025 [33] (43/66 Moderate). | Supportive but heterogeneous. Enalapril: ADRB2 rs1042714 GC AOR 2.31 [29]. Amlodipine: ACE rs4291 AA AOR 0.23 [30]. RHTN: CYP3A5 CC protective; NEDD4L AA and ADRB1 GG susceptibility [31]; NOS3 rs3918188 A/A protective (aOR 0.13) [33]. HCTZ: GNB3 rs1025450259 associated with BP reduction [32]. | Drug (enalapril, amlodipine, HCTZ, RHTN panels), ancestry subgroup, outcome definition (controlled BP vs. RHTN), cross-sectional vs. case-control design, variant panel scope, multiple-comparison correction. | Directionally supportive if lower-quality studies excluded; drug-specific certainty weakens without the Good-rated Katsukunya 2024 [31]. No RCT in this domain; cross-sectional designs limit causal inference. |
| **S5. SLCO1B1 – statin-induced myotoxicity** | 1 study: Haldar 2025 [34] (Q-Genie 43/66 Moderate; JBI Analytical Cross-Sectional: Seek further info). Biobank/EHR-linked; n=527,500 for bilirubin; 578 myotoxicity cases. | Suggestive. rs59502379 OR=2.85 (95%CI 1.08–7.52) for myotoxicity; bilirubin p=7.4×10⁻¹². Variant enriched in sub-Saharan African-ancestry populations; 31.5% of UAE CVD patients carried myopathy-risk SLCO1B1 variant [39]. rs77271279 myotoxicity OR=1.75 (95%CI 0.62–4.73) non-significant. | Between-study heterogeneity not assessable (single study). Within-study: EHR phenotype ascertainment, bilirubin as proxy biomarker, ancestry similarity classification, statin type/dose heterogeneity. | Excluding this single study removes all SLCO1B1 myotoxicity synthesis. Conclusions must be framed as biologically plausible and suggestive, not definitive. Replication in African-ancestry LMIC cohorts essential. |
| **S6. BDNF / ATP2A2 – empagliflozin response in HFrEF** | 1 study: Ain 2025 [35] (Q-Genie 35/66 Moderate). Prospective cohort, Pakistan; n=99 genotyped. No control arms. | Suggestive/hypothesis-generating. BDNF rs6265 CC: BNP-response OR=7.70; EF-response OR=5.97. ATP2A2 rs1860561 GG: EF-response OR=5.97. CIs not reported; p-values: BNP p<0.001, EF p=0.028. First LMIC pharmacogenomic characterization of an SGLT2 inhibitor. | Not assessable between studies. Within-study: surrogate endpoints (BNP, LVEF), no comparator arm, limited covariate adjustment detail, missing precision data (no 95% CIs). | Strict precision criterion would suspend interpretation; directional signal hypothesis-generating only. Replication with complete precision reporting and a comparator arm required before clinical translation. |
| **S7. Population PGx profiling – actionable variant burden** | 6 sources: Muyambo 2022 [23] (Q-Genie 27/66 Poor), Ndadza 2021 [24] (29/66 Poor), Runcharoen 2021 [36] (JBI: Seek further info), Sherman 2024 [37] (JBI: Include), Acosta-Monterrosa 2025 [38] (JBI: Seek further info), Al-Mahayri 2022 [39] (JBI: Exclude). | Supportive. 98.2% of individuals had ≥1 non-typical PGx phenotype [37]; 28 SNPs more frequent in African-leaning vs. European-leaning Colombian populations [38]; DPYD IM frequencies differed 69.8-fold across super-populations [37]; 96% of UAE CVD patients received actionable PGx recommendation [39]. Ancestry-specific variant architecture requires population-aware implementation. | Platform (panel vs. WGS vs. star-allele), database vs. cohort sampling, variant-calling framework, ancestry definition, actionability metric, star-allele definitions, reference genome version. | Cross-population evidence from SEAPharm [36], 1000 Genomes [37], and Colombia [38] supports the equity conclusion even excluding the JBI-Excluded Al-Mahayri [39] and the two Poor-rated Southern African profiling studies [23,24]. |
| **S8. Implementation, stakeholder barriers, and adoption** | 3 studies: Che Mustapa 2020 [40] (n=421, Malaysia; JBI Analytical Cross-Sectional: Include), Kamp 2022 [41] (n=109, South Africa; JBI: Seek further info), Salas-Hernández 2023 [42] (n=106, 17 LAC countries; JBI: Seek further info). | Convergent. Top barriers: need for clinical guidelines 9.06/10 [42]; high cost (89% of clinicians) [41]; lack of cost-effectiveness evidence 8.96/10 [42]. Adoption predictors: perceived benefits β=0.371, trust β=0.312, engagement β=0.272 [30]. 78% of SA clinicians had low PGx knowledge [41]. CYP2C19/clopidogrel ranked most useful gene–drug pair [42]. | Stakeholder group (clinicians vs. patients/providers), country, health-system context, measurement construct (PLS-SEM path coefficients vs. percentage agreement vs. barrier ranking), survey instrument validation. | Barrier profile directionally stable excluding weaker-appraisal studies. Removing Salas-Hernández [42] loses multi-country LAC representation; removing Che Mustapa [40] loses quantitative adoption-determinant data. Core conclusion unchanged. |

Abbreviations: ICER, incremental cost-effectiveness ratio; BCR, benefit-to-cost ratio; WTP, willingness-to-pay threshold; QALY, quality-adjusted life year; TTR, time in therapeutic INR range; TRT, time to reach therapeutic INR; RHTN, resistant hypertension; LOF, loss-of-function; LMIC, low- and middle-income country; LAC, Latin America and the Caribbean; PLS-SEM, partial least squares structural equation modelling; DB, database; CEA, cost-effectiveness analysis; CBA, cost-benefit analysis; WGS, whole-genome sequencing.

## Overall Robustness, Cross-Synthesis Interpretation, and GRADE Certainty

The strongest conclusions across the eight syntheses were those supported by converging evidence from multiple study designs or populations: the economic benefit of CYP2C19-guided antiplatelet therapy in Asian stroke/TIA settings (S1; two models, one allele-frequency study); the pharmacogenomic relevance of CYP2C9 and VKORC1 for warfarin dose variability and anticoagulation quality in South Asian and sub-Saharan African populations (S2; one GWAS, one dose-determinant study, and one RCT); and the need for ancestry-aware PGx implementation in LMICs (S7; six convergent sources). Findings of only one eligible trial SLCO1B1 myotoxicity (S5), BDNF/ATP2A2 empagliflozin response (S6) should be considered intriguing and hypothesis-generating, not practice-changing. The economic evidence for warfarin-related pharmacogenomics was the most context-dependent of any area (S3), with the direction of cost-effectiveness findings reliant on local genotyping test costs, adverse-event probability assumptions, and payer perspective.

The strongest cross-cutting sources of heterogeneity population ancestry, clinical indication, study design, outcome definition, genotyping platform, time horizon, and health-system cost structure were substantial and clinically meaningful across all syntheses. The decision not to conduct a statistical meta-analysis was therefore appropriate and pre-specified. Qualitative sensitivity analysis across syntheses showed that most directional conclusions were directionally stable when lower-quality studies were excluded, with the exceptions noted above for single-study syntheses and for the context-sensitive economic evaluations.

GRADE certainty of evidence assessments, utilising the SWiM approach applied to narrative synthesis without pooled effect estimates, are reported in the additional Certainty of Evidence tables (Supporting Information). Anticoagulation control (TTR) was rated as Moderate certainty for the primary outcome in the warfarin RCT (S2; one RCT rated ‘Some concerns’). All remaining outcomes were rated as Very Low certainty, reflecting the predominance of observational evidence, indirect comparisons, imprecision and in several cases single-study designs. This GRADE profile is consistent with the early-phase implementation evidence base for pharmacogenomics-guided cardiovascular therapy in LMICs and highlights the priority of undertaking appropriately powered, prospective clinical studies in these populations.-
